# Assessing Potential Habitat Suitability of the Endangered Endo-Holoparasitic *Sapria himalayana* and Its Multiple Hosts in China Under Global Warming

**DOI:** 10.3390/plants15040574

**Published:** 2026-02-11

**Authors:** Weiyi Hang, Yan Li, Guangfu Zhang

**Affiliations:** 1 Key Laboratory of Biodiversity and Biotechnology, School of Life Sciences, Nanjing Normal University, Nanjing 210023, China; 241202166@njnu.edu.cn; 2 Nanjing Institute of Environmental Science, Ministry of Ecology and Environment of the People’s Republic of China, Nanjing 210042, China; liyan@nies.org

**Keywords:** environmental variable, global change, habitat suitability, MaxEnt modeling, niche overlap, China

## Abstract

Global warming severely threatens parasitic plants worldwide. However, little is known about how a parasite with multiple hosts responds to climate change in its distribution. *Sapria himalayana* is an endangered endo-holoparasite, obligately parasitizing *Tetrastigma* species. We employed MaxEnt to predict suitable habitats for *S. himalayana* and its five hosts, and determined key environmental factors. Then, we calculated niche overlaps for the five parasite-host pairs. Currently, it covers a suitable area of 1.35 × 10^4^ km^2^, accounting for 0.14% of China’s total territory. Temperature-related variables were identified as the key factors shaping potential distribution for this parasite and three hosts (i.e., *T. planicaule*, *T. obovatum*, and *T. cruciatum*), while precipitation-related ones were identified for the other hosts (i.e., *T. obtectum* and *T. serrulatum*). Collectively, the five pairs presented low niche overlaps under current and future scenarios. While *S. himalayana* will increase by 37.78% in future suitable habitat, the two host categories show contrasting trends in potential habitat shifts. Divergent climatic sensitivities across host species, along with parasite–host suitability mismatches, could shape the survival and distribution of *S. himalayana*. Consequently, this research offers valuable insights for the conservation of *S. himalayana* in China, highlighting the necessity of safeguarding its distinct hosts under global warming.

## 1. Introduction

Global warming is altering temperature and precipitation patterns worldwide, thereby affecting species’ growth, reproduction, and distribution [1,2]. Parasitic plants are impacted by global warming more severely compared to non-parasitic plants [3]. The impact of climate change on parasitic plants operates through two primary pathways. Firstly, it can directly modify their habitat conditions, necessitating range shifts [4,5]. Secondly, because parasitic plants depend functionally on hosts via haustoria for water and nutrient acquisition [6], their distribution is further constrained by the responses of their host species to climate change. According to the host-quality hypothesis, the distribution of parasitic plants is limited by the quality of their hosts—specifically, the availability of resources such as water and nutrients that are typically limiting the hosts themselves [7]. Consequently, global warming can also indirectly impact the survival of parasitic plants by changing the strength of host–parasite interactions [8]. Endangered plants often have characteristics of small population sizes, narrow geographic distributions, and low genetic diversity [9]. For endangered parasitic plants, their distribution is further restricted by the environmental requirements of their hosts, making them more sensitive to climate change. Based on the definition by [10], parasitic plants can be classified into four functional categories: root hemiparasites, root holoparasites, stem parasites, and endophytic parasites. Endoparasitic plants exhibit a suite of distinct adaptations, including obligate parasitism with immediate haustorial development, a dominant endophytic phase, a vegetative body reduced to a mycelium-like structure, and transient exophytes only during flowering and fruiting [10,11]. Consequently, endangered species within this group are likely to be disproportionately vulnerable to global warming.

Currently, numerous studies have predicted the distribution of parasitic plants [12,13,14], yet only a few have simultaneously addressed how both parasitic plants and their hosts may respond to future climate change [15,16]. In fact, hosts play a significant role in shaping the distribution and survival of parasitic plants, particularly for those with high host specificity [3]. For parasitic plants that depend on obligate hosts, the number of hosts can range from one to several (*n* ≥ 2). However, current research has largely focused on single-host systems, with multi-host parasites receiving comparatively little attention [5,17].

Endophytic parasites represent a small subset of all parasitic plants, restricted to the families Apodanthaceae, Cytinaceae, Rafflesiaceae, and several genera of Santalales. Most of these are obligate parasites and can rely on multiple host species [10,18]. For example, all species of the genus *Rafflesia* (Rafflesiaceae) are obligate parasites on vines of the genus *Tetrastigma* and are known to utilize multiple species within this genus as hosts [19,20,21].

*Sapria himalayana* Griff. is a fleshy herb from the family Rafflesiaceae. As an obligate endoparasite, it lacks leaves, roots, and stems, and persists predominantly as thread-like filaments within its host tissues. The only emergent stage occurs during flowering, which presents ten bright red bracts marked with sulphur-yellow spots [22,23]. This species parasitizes several members of the genus *Tetrastigma* (Vitaceae) [24]. Although earlier records reported its occurrence on *Vitis* species [25], such accounts are likely attributable to historical misidentification of certain *Tetrastigma* taxa as *Vitis* in the same family [26]. Current research on *S. himalayana* primarily focuses on genetic [27], biochemical [28], and physiological [29] aspects, but little is known about its potential distribution.

*S. himalayana* has a narrow distribution in China. According to *Flora of China* (FOC), it only occurs in Yunnan Province and Xizang Autonomous Region (i.e., Tibet) [25], with no updated reports from other provinces in the country [18]. Based on the existing literature, five *Tetrastigma* species are recognized as hosts for *S. himalayana*: *Tetrastigma planicaule* (Hook.) Gagnep., *T. obtectum* (Wall.) Planch., *T. serrulatum* (Roxb.) Planch., *T. obovatum* Gagnep., and *T. cruciatum* Craib & Gagnep. [30,31,32,33,34]. Furthermore, according to *Flora of China*, all these climbers occur in Yunnan, the primary distribution area of *S. himalayana* [35]. Therefore, these five species are considered the major hosts of *S. himalayana*.

Nowadays, *S. himalayana* is threatened primarily by habitat destruction and illegal excavation within its known range. Such a threat is exacerbated by the species’ inherent reproductive challenges—including high bud mortality and strongly skewed sex ratio—coupled with its dependence on specific host plants [36,37,38]. These factors jointly render the species endangered in China. Therefore, this species has been listed as a Grade II National Key Protected Wild Plant in China and categorized as a “Vulnerable (VU)” species in the IUCN Red List [39]. However, it remains unclear what the potential geographic distribution of *S. himalayana* is, and how the endangered parasite and its five primary hosts respond in the context of climate change.

This study employed a species distribution modeling framework (MaxEnt) to project the potential geographic distributions of *S. himalayana* and its five obligate host species, utilizing known occurrence data and environmental variables. The work aimed to (1) identify the key environmental factors and shared drivers shaping their distributions, (2) predict shifts in their suitable habitats under current and future climate scenarios, and (3) quantify the niche overlap between each parasite–host pair across these scenarios. Ultimately, our findings are intended to inform conservation strategies for *S. himalayana* and other endangered multi-host endophytic parasites.

## 2. Results

### 2.1. Model Performance and Key Environmental Factors

AUC values of prediction models for *Sapria himalayana* and its five hosts (i.e., *Tetrastigma planicaule*, *T. obtectum*, *T. obovatum*, *T. cruciatumc* and *T. serrulatum*) under different climate scenarios were all greater than 0.90 (Table S1), indicating excellent model performance for all species. In addition, TSS values of the six species across different climate scenarios were all above 0.75 (Table S1). Specifically, *S. himalayana* and *T. cruciatum* had TSS values exceeding 0.9; *T. planicaule* and *T. serrulatum* had TSS values exceeding 0.8; while *T. obtectum* and *T. obovatum* had TSS values exceeding 0.75 (Table S1). Generally, a TSS value above 0.75 suggests a model with strong predictive performance [40]. Overall, these results indicated high accuracy and credibility of MaxEnt models for each species under both current and future climate scenarios.

The results showed that extrapolation risks were collectively much lower for both *S. himalayana* and its five hosts, although there were certain differences in this respect for these six species (Table 1). For *S. himalayana*, its future average proportion of pixels with multivariate environmental similarity surface (MESS) value < 0 was 1.6512%. For the five hosts, *T. planicaule* had the highest average proportion of pixels with MESS < 0 under future conditions (i.e., 2.5877%). For *T. obtectum*, *T. obovatum*, *T. cruciatum*, and *T. serrulatum*, their corresponding proportions were 0.7791%, 0.8555%, 1.1138%, and 1.1750%, respectively (Table 1). Moreover, for both *S. himalayana* and its five hosts, the proportion of pixels with MESS < 0 varied noticeably across different future climate scenarios (Table 1).

Among the six species, *S. himalayana* had the greatest extent of niche unfilling, reaching 90.38%, followed by *T. planicaule*, *T. obtectum*, and *T. cruciatum* with percentages of 65.00%, 63.76%, and 60.61%, respectively. In contrast, *T. serrulatum* only had the niche unfilling of 37.65%; *T. obovatum* had the lowest value of 18.18% (Table 2).

For each species, the top five environmental variables, whose cumulative contribution exceeded 90%, were identified as key predictors (Table 3). For *S. himalayana*, the key factors affecting its potential distribution were isothermality (bio3, 88.4%), temperature seasonality (bio4, 4.8%), annual mean temperature (bio1, 3.3%), precipitation of warmest quarter (bio18, 1.2%) and aspect (0.6%), together accounting for 98.3% of the total.

For *T. planicaule*, the key factors affecting its potential distribution were mean temperature of coldest quarter (bio11, 67.4%), annual precipitation (bio12, 16.2%), temperature seasonality (bio4, 5.7%), human influence (HI, 4.4%) and mean temperature of wettest quarter (bio8, 1.6%), totaling 95.3%.

For *T. obtectum*, the key factors affecting its potential distribution were annual precipitation (bio12, 40.1%), min temperature of coldest month (bio6, 19.9%), precipitation of driest month (bio14, 15.2%), elevation (12.7%) and human influence (HI, 7.8%), totaling 92.8%.

For *T. obovatum*, the key factors affecting its potential distribution were min temperature of coldest month (bio6, 51.4%), isothermality (bio3, 17.9%), human influence (HI, 11.2%), annual precipitation (bio12, 7.8%) and precipitation of driest quarter (bio17, 7.5%), totaling 95.8%.

For *T. cruciatum*, the key factors affecting its potential distribution were temperature seasonality (bio4, 72.9%), isothermality (bio3, 16.1%), annual precipitation (bio12, 3.2%), precipitation of wettest month (bio13, 2.2%) and human influence (HI, 2.2%), totaling 96.6%.

For *T. serrulatum*, the key factors affecting its potential distribution were precipitation of driest quarter (bio17, 33.8%), temperature seasonality (bio4, 33.7%), elevation (11.4%), annual precipitation (bio12, 9.3%) and human influence (HI, 6.1%), totaling 94.3%.

Three temperature-related variables (i.e., bio3, bio4, and bio1) collectively accounted for 96.5% of the explained contribution among the five key factors shaping the distribution of *S. himalayana*, underscoring their dominant role. Likewise, temperature-related variables constituted the primary explanatory factors for *T. planicaule*, *T. obovatum*, and *T. cruciatum*, with cumulative contributions of 74.7%, 69.3%, and 89.0%, respectively. In contrast, precipitation exerted a greater influence on distribution for *T. obtectum* and *T. serrulatum*, with their precipitation-related variables contributing 55.3% and 43.1% in total, respectively (Table 3).

### 2.2. Potential Suitable Distribution of Sapria himalayana and Its Five Hosts

**Figure 1:**
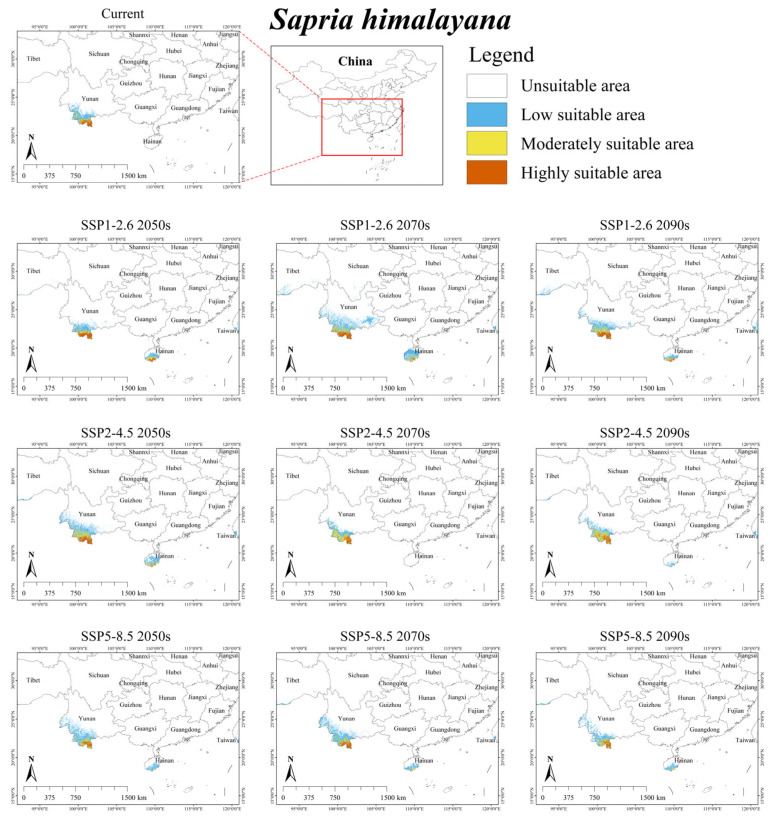
Predicted suitable habitat distribution of *Sapria himalayana* under different climate scenarios in China.

Under the current climate scenario, suitable habitat (moderately and highly suitable areas) for *S. himalayana* was mainly located in southern Yunnan (Figure 1), with a total area of 1.35 × 10^4^ km^2^, accounting for approximately 0.14% of China’s territory. Across the nine future climate scenarios, the suitable habitat for *S. himalayana* was projected to expand by an average of 37.78%, reaching a total area of 1.86 × 10^4^ km^2^ (Table 4). Only under SSP5-8.5 2050s and SSP5-8.5 2090s did its suitable habitat show a slight decrease of 4.44% and 0.74%, respectively.

**Figure 2:**
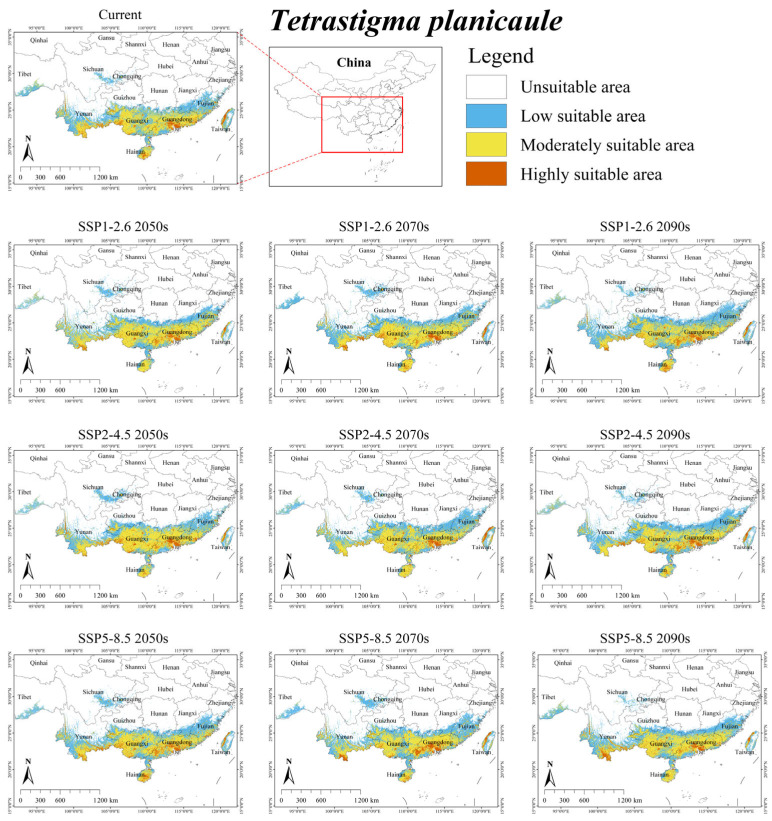
Predicted suitable habitat distribution of *Tetrastigma planicaule* under different climate scenarios in China.

Under the current climate scenario, the suitable habitat for *T. planicaule* was mainly distributed in southeastern coastal regions of China (Guangxi, Guangdong, Hainan, southern Fujian, and western Taiwan), as well as southwestern China (western and southern Yunnan, southern Guizhou) (Figure 2). The total area was 36.68 × 10^4^ km^2^, accounting for approximately 3.82% of China’s territory (Table 4). Additionally, its overlap suitable area with *S. himalayana* was 0.93 × 10^4^ km^2^, accounting for 68.89% of the total suitable habitat for *S. himalayana* (Table S2).

Under future climate scenarios, the suitable habitat area for *T. planicaule* was predicted to expand by an average of 2.51%, reaching a total area of 37.60 × 10^4^ km^2^ (Table 4). Only under SSP1-2.6 2090s and SSP2-4.5 2090s did the suitable habitat area show a slight decrease of 2.86% and 1.66%, respectively. Furthermore, its overlap with the suitable area of *S. himalayana* was projected to increase by an average of 34.41%, reaching a total area of 1.25 × 10^4^ km^2^ (Table S2).

**Figure 3:**
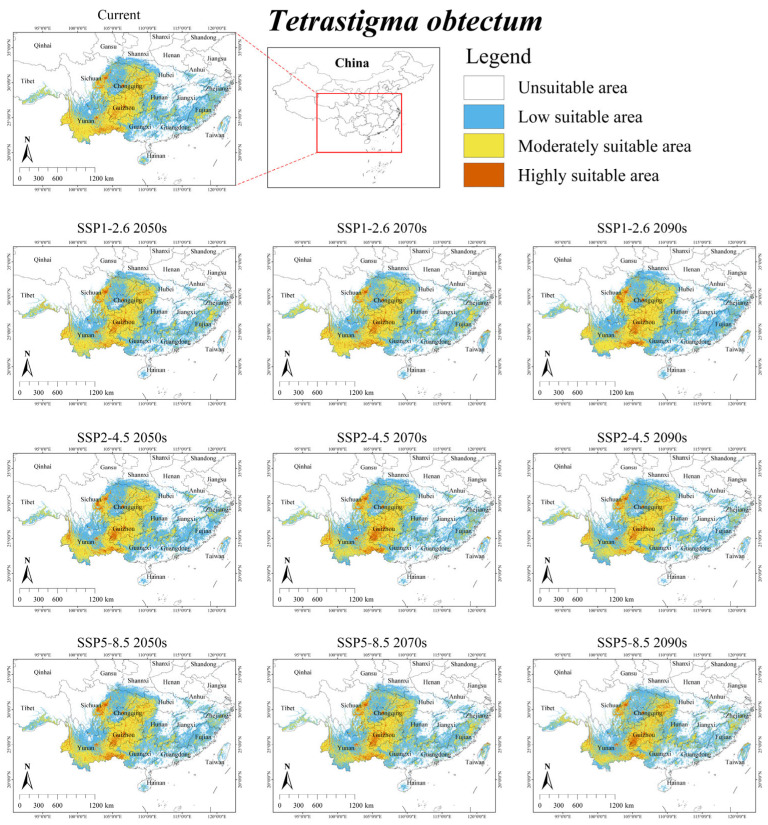
Predicted suitable habitat distribution of *Tetrastigma obtectum* under different climate scenarios in China.

Under the current climate scenario, the suitable habitat for *T. obtectum* was mainly distributed in southwestern China (Chongqing, Guizhou, eastern and southern Sichuan, southeastern Tibet, Yunnan) and surrounding provinces (southern Shaanxi, southern Henan, western Guangxi, western Hubei, western Hunan), with several fragmented suitable habitats scattered in southeastern coastal regions (Fujian, Guangdong, Hainan, Zhejiang, western Taiwan) (Figure 3). The total area was 71.70 × 10^4^ km^2^, accounting for approximately 7.47% of China’s territory (Table 4). Additionally, its overlap suitable area with *S. himalayana* was 0.89 × 10^4^ km^2^, constituting 65.93% of the latter’s total suitable habitat (Table S2).

Under future climate scenarios, the suitable habitat area for *T. obtectum* was predicted to reduce by an average of 3.26%, dropping to an area of 69.36 × 10^4^ km^2^ (Table 4). Only under SSP1-2.6 2070s did the suitable habitat area increase by 6.60%. Furthermore, its overlap with the suitable area of *S. himalayana* was projected to decrease by an average of 20.22%, dropping to an area of 0.71 × 10^4^ km^2^ (Table S2).

**Figure 4:**
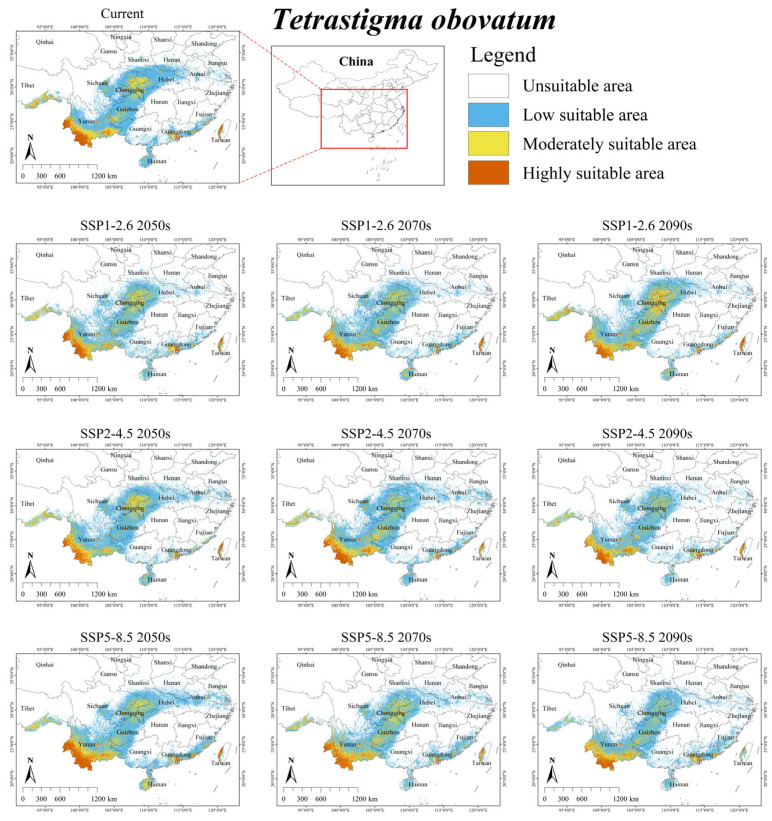
Predicted suitable habitat distribution of *Tetrastigma obovatum* under different climate scenarios in China.

Under the current climate scenario, the suitable habitat for *T. obovatum* was mainly distributed in southwestern China (Chongqing, Guizhou, eastern Sichuan, southeastern Tibet, Yunnan) and surrounding provinces (western Hubei), with several suitable habitats distributed in southeastern coastal regions (central Guangdong, northern Hainan, southwestern Fujian, western Taiwan) (Figure 4). The total area was 30.42 × 10^4^ km^2^, accounting for approximately 3.17% of China’s territory (Table 4). Additionally, its overlap with the suitable area of *S. himalayana* was 1.35 × 10^4^ km^2^, accounting for 100% of the total suitable habitat area for the endangered parasite (Table S2).

Under future climate scenarios, the suitable habitat for *T. obovatum* was predicted to expand by an average of 0.95%, reaching an area of 30.71 × 10^4^ km^2^ (Table 4). Across all SSP2-4.5 scenarios, the suitable habitat area of *T. obovatum* decreased, with an average decline of 6.28%. Under the SSP1-2.6 2070s and SSP5-8.5 2090s, the suitable habitat area for *T. obovatum* also showed a decrease of 3.52% and 13.02%, respectively. Furthermore, its overlap with the suitable area of *S. himalayana* was projected to increase by an average of 22.96%, reaching a total area of 1.66 × 10^4^ km^2^ (Table S2).

**Figure 5:**
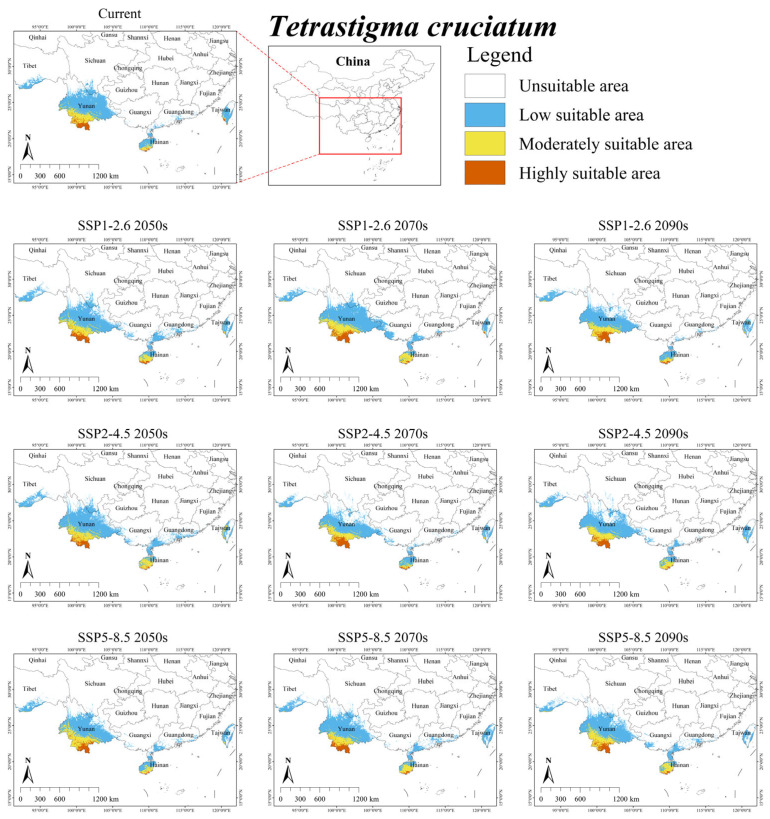
Predicted suitable habitat distribution of *Tetrastigma cruciatum* under different climate scenarios in China.

Under the current climate scenario, the suitable habitat for *T. cruciatum* was mainly distributed in southern Yunnan, southern Hainan, and southwestern Taiwan (Figure 5). The total area was 7.35 × 10^4^ km^2^, accounting for approximately 0.77% of China’s territory (Table 4). Additionally, its overlap with the suitable area of *S. himalayana* was 1.35 × 10^4^ km^2^, accounting for 100% of the total suitable habitat area for the parasite (Table S2).

Under future climate scenarios, the suitable habitat area for *T. cruciatum* was predicted to expand by an average of 8.16%, reaching a total area of 7.95 × 10^4^ km^2^ (Table 4). Under four future scenarios, the suitable habitat area of *T. cruciatum* exhibited a slight decrease (i.e., SSP1-2.6 2090s, 5.99%; SSP2-4.5 2070s, 1.77%; SSP2-4.5 2090s, 1.36%; SSP5-8.5 2070s, 0.95%). Furthermore, its overlap with the suitable area of *S. himalayana* was projected to increase by an average of 36.30%, reaching a total area of 1.84 × 10^4^ km^2^ (Table S2).

**Figure 6:**
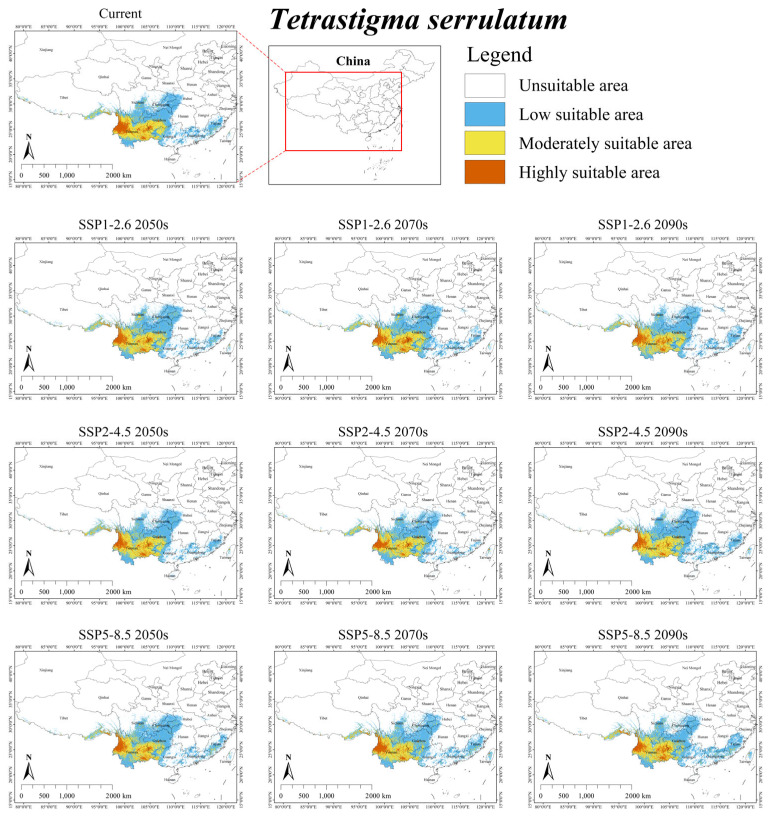
Predicted suitable habitat distribution of *Tetrastigma serrulatum* under different climate scenarios in China.

Under the current climate scenario, the suitable habitat for *T. serrulatum* was mainly distributed in southwestern China (southeastern Guizhou, southern Sichuan, southeastern Tibet, Yunnan) and surrounding provinces (western Guangxi), with several fragmented suitable habitats scattered in southeastern Fujian (Figure 6). The total area was 38.09 × 10^4^ km^2^, accounting for approximately 3.97% of China’s territory (Table 4). Additionally, its overlap with the suitable area of *S. himalayana* was 0.04 × 10^4^ km^2^, accounting for only 2.96% of the total suitable habitat area for the parasite (Table S2).

Under future climate scenarios, the suitable habitat area for *T. serrulatum* was predicted to reduce by an average of 4.25%, dropping to an area of 36.47 × 10^4^ km^2^ (Table 4). Only under SSP1-2.6 2050s and SSP1-2.6 2070s did the suitable habitat area of *T. serrulatum* show a slight increase of 2.86% and 2.13%, respectively. Furthermore, its overlap suitable area with *S. himalayana* was projected to increase by an average of 272.00%, reaching a total area of 0.15 × 10^4^ km^2^ (Table S2).

Overall, the endangered parasite and its five hosts showed divergent future trends in suitable habitat, and they could be divided into two groups: increasing type (*S. himalayana*, *T*. *planicaule*, *T. obovatum*, and *T. cruciatum*) and decreasing type (*T. obtectum* and *T. serrulatum*).

### 2.3. Future Centroid Shifts of Sapria himalayana and Its Five Hosts

**Figure 7:**
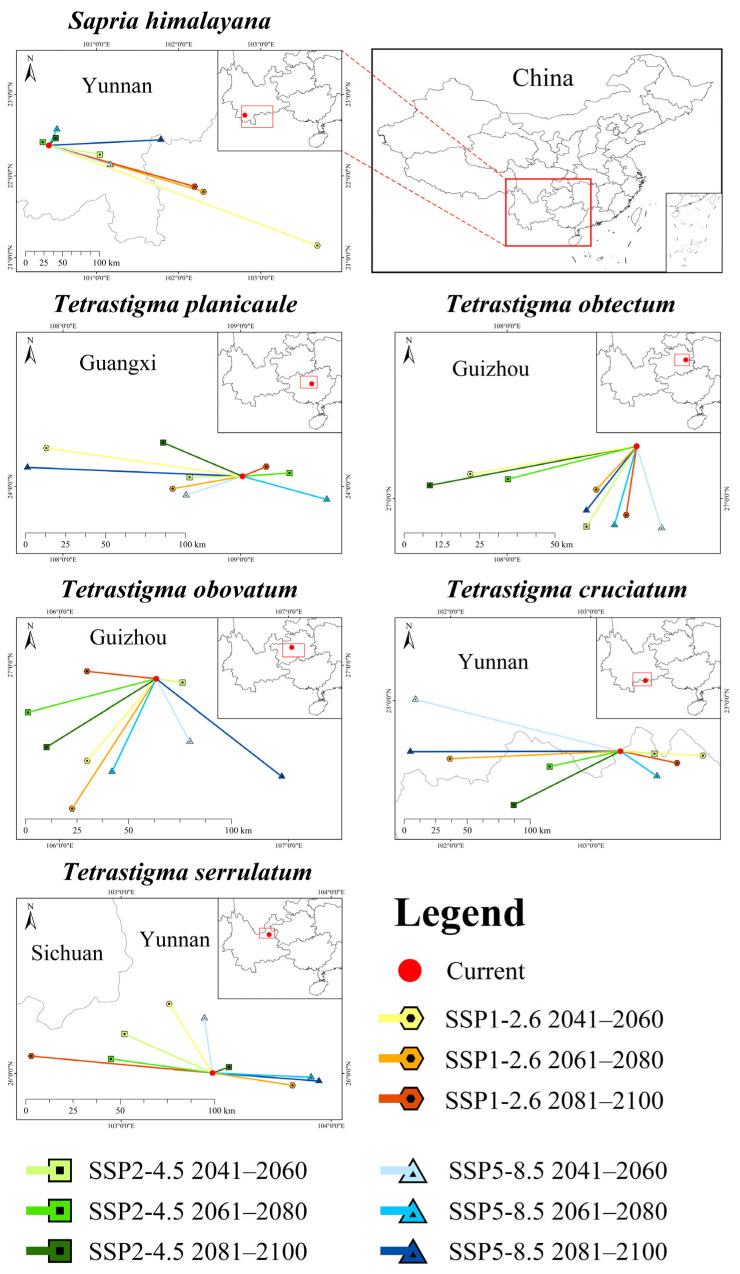
Shift of the core distribution of *Sapria himalayana* and its five hosts under nine future climate scenarios in China. For each species, the centroid shift map shows a close-up view of the corresponding red rectangle in the upper-right corner.

The suitable habitat centroid for *S. himalayana* under the current climate was located in the southern part of Yunnan Province (22.37° N, 100.42° E). Under future climate scenarios, its centroid was projected to shift predominantly southeastward (Figure 7). The longest shifting distance was observed under SSP1-2.6 2050s, with a southeastward movement of 363.67 km to 21.15° N, 103.69° E.

The centroid of *T. planicaule* under the current climate was located in the central part of Guangxi Province (24.06° N, 109.01° E). Under future climate scenarios, no noticeable trend was identified in its centroid shift direction (Figure 7). The longest shifting distance occurred under SSP5-8.5 2090s, with a northwestward movement of 122.90 km to 24.11° N, 107.80° E.

The centroid of *T. obtectum* under the current climate was located in the eastern part of Guizhou Province (27.16° N, 108.39° E). Under future climate scenarios, its centroid was projected to shift predominantly southwestward (Figure 7). The longest shifting distance occurred under SSP2-4.5 2090s, with a southwestward movement of 62.47 km to 27.04° N, 107.77° E.

The centroid of *T. obovatum* under the current climate was located in the central part of Guizhou Province (26.94° N, 106.42° E). Under future climate scenarios, its centroid was projected to shift predominantly southwestward (Figure 7). The longest shifting distance occurred under SSP1-2.6 2070s, with a southwestward movement of 73.13 km to 26.37° N, 106.05° E.

The centroid of *T. cruciatum* under the current climate was located in the southeastern part of Yunnan Province (22.64° N, 103.21° E). Under future climate scenarios, no noticeable trend was identified in its centroid shift direction (Figure 7). The longest shifting distance occurred under SSP5-8.5 2050s, with a northwestward movement of 155.59 km to 23.02° N, 101.75° E.

The centroid of *T. serrulatum* under the current climate was located in the northeastern part of Yunnan Province (26.00° N, 103.43° E). Under future climate scenarios, its centroid was projected to shift predominantly northwestward (Figure 7). The longest shifting distance occurred under SSP1-2.6 2090s, with a northwestward movement of 86.69 km to 26.08° N, 102.57° E.

In brief, the five hosts exhibited different future centroid shifts and can be categorized into three types: shifting southwestward (i.e., *T. obtectum* and *T. obovatum*), shifting northwestward (i.e., *T. serrulatum*), and shifting in various directions (i.e., *T. planicaule* and *T. cruciatum*). Accordingly, these hosts differed from *S. himalayana* in centroid shift.

### 2.4. Niche Overlap of Sapria himalayana and Its Five Hosts

The niche overlap indices *D* and *I* between *S. himalayana* and *T. planicaule* under the current scenario were 0.1299 and 0.5118, respectively (Table 5), indicating a very limited niche overlap between them. Under future scenarios, both *D* and *I* values were predicted to increase, with their mean values reaching 0.1923 and 0.5493, respectively (Table 5). Notably, the level of niche overlap still remained in the “very limited overlap” category.

The niche overlap indices *D* and *I* between *S. himalayana* and *T. obtectum* under the current scenario were 0.1435 and 0.4899, respectively (Table 5), indicating a very limited niche overlap. Under future scenarios, both *D* and *I* values were predicted to increase, with their mean values reaching 0.1965 and 0.5093, respectively (Table 5). Notably, the level of niche overlap still remained in the “very limited overlap” category.

The niche overlap indices *D* and *I* between *S. himalayana* and *T. obovatum* under the current scenario were 0.2162 and 0.5851, respectively (Table 5), indicating a low niche overlap. Under future scenarios, both *D* and *I* values were predicted to increase, with their mean values reaching 0.2925 and 0.6265, respectively (Table 5). Notably, the level of niche overlap still remained in the “low overlap” category.

The niche overlap indices *D* and *I* between *S. himalayana* and *T. cruciatum* under the current scenario were 0.4413 and 0.7960, respectively (Table 5), indicating a moderate niche overlap level. Under future scenarios, both *D* and *I* values were predicted to increase, with their mean values reaching 0.5556 and 0.8498, respectively (Table 5). Notably, the level of niche overlap still remained in the “moderate overlap” category.

The niche overlap indices *D* and *I* between *S. himalayana* and *T. serrulatum* under the current scenario were 0.1903 and 0.5300, respectively (Table 5), indicating a very limited niche overlap. Under future scenarios, both *D* and *I* values were predicted to increase, with their mean values reaching 0.2601 and 0.5802, respectively (Table 5). In this case, the level of niche overlap increased to the “low overlap” category.

Taken together, both niche overlap indices revealed consistent patterns across the five species pairs. They can be classified into three categories: moderate overlap (i.e., *S. himalayana* vs. *T. cruciatum*), low overlap (i.e., *S. himalayana* vs. *T. obovatum*; *S. himalayana* vs. *T. serrulatum*), and very limited overlap (i.e., *S. himalayana* vs. *T. planicaule*; *S. himalayana* vs. *T. obtectum*) (Table 5).

## 3. Discussion

### 3.1. Model Evaluation and Key Factors

The potential distributions of the endangered *S. himalayana* and its five host species were projected using MaxEnt models. Prior to final modeling, each species-specific model was optimized through parameter tuning of the regularization multiplier (RM) and feature class (FC) to enhance robustness and mitigate overfitting. The optimized models demonstrate high predictive performance, as reflected in strong evaluation metrics. For *S. himalayana*, both the AUC and TSS values indicate excellent model fit (Table S1), and its predicted current distribution aligns closely with existing occurrence records. Similarly, all host plant models performed reliably, with AUC and TSS scores consistently within the range indicative of high accuracy (Table S1). These results confirm that the tuned models are suitable for assessing current and future habitat suitability across the targeted species. Furthermore, our results show that the MESS values for most of the predicted areas are positive (Table 1), indicating that these areas are similar to the model calibration area in environmental conditions, and that the projection outcomes are mainly based on the simulation of the observed environmental range.

Our model indicates that the top three key factors influencing the current distribution of *S. himalayana* are bio3, bio4, and bio1, with their combined contribution reaching 96.5% (Table 3). It is noteworthy that all three factors are temperature-related climatic variables, suggesting that temperature, rather than topography or anthropogenic influence, is the primary limiting factor for the endangered holoparasite in distribution. *S. himalayana* belongs to the genus *Sapria* from the family Rafflesiaceae, and now this genus only comprises four species [41]. Following Wu Zhengyi’s classification for the genus areal-types of Chinese seed plants, this genus exhibits a tropical Asian distribution pattern, with its members spanning from tropical India to southern China [18]. According to *Flora of China*, there is just one species, namely *S. himalayana*, in China. It is confined to Zayü of Tibet, as well as Lancang, Mengla, and Jinghong of Yunnan Province, China [25,38]. The known records of this species align with the outcome of our MaxEnt modeling (Figure 1). Currently, only some studies have analyzed the ecological requirements of *S. himalayana*. Chaisung et al. [42] reported that its three populations in Chiang Mai Province, Thailand, were affected by soil temperature, air temperature, and light intensity, and they contended that temperature affected its population mortality rate, because lower temperatures corresponded to higher mortality. Bänziger et al. [29] discovered that even during the dry season, the humidity in the perianth tube of this species in Chiang Mai Province, northern Thailand, remained consistently close to saturation. This was because the water transpired from the perianth tube could readily be replenished from the host *Tetrastigma*. As a result, it seems likely that precipitation may not be a key factor limiting its population distribution therein. Combined with our model projections, the results indicate that *S. himalayana* favors warm, humid climates and that its distribution is more strongly constrained by temperature than by other variables.

The five host species of *S. himalayana* can be divided into two categories based on their primary influencing factors. *T*. *planicaule*, *T. obovatum*, and *T. cruciatum* are mainly influenced by temperature-related climatic variables in distribution, while *T. obtectum* and *T. serrulatum* are primarily affected by precipitation-related climatic variables (Table 3). Key environmental factors shared between *S. himalayana* and its hosts varied among species. Bio3 emerged as a common key factor for *T. obovatum* (17.9%) and *T. cruciatum* (16.1%) in relation to *S. himalayana* (88.4%). Also, bio4 was identified as a shared factor for *T. serrulatum* (33.7%), *T. planicaule* (5.7%) and *S. himalayana* (4.8%). In contrast, *T. obtectum* and *S. himalayana* have no key common factors (Table 3). Furthermore, a notable pattern emerged regarding future habitat trends between two host groups. Host species whose distribution was primarily influenced by temperature-related factors exhibited an expansion in suitable habitat, whereas those governed mainly by precipitation-related factors showed a contraction. This may suggest that although both *S. himalayana* and its five hosts are primarily influenced by climatic factors, there is a considerable difference in the key climate factors for these five hosts. Furthermore, the Human Influence (HI) is identified as a key common factor shaping the suitable distribution for the five hosts, rather than for the parasite, highlighting a greater anthropogenic pressure on these hosts.

### 3.2. Suitable Range and Future Change for Sapria himalayana and Its Hosts

This study provides the first delineation of the suitable habitat for *S. himalayana* in China under current climatic conditions, covering approximately 1.35 × 10^4^ km^2^ (0.14% of China’s land area). Projections suggest this area may expand to 1.86 × 10^4^ km^2^ in future climates (Table 4). Notably, although some parts of Taiwan fall into the suitable habitat range of *S. himalayana* and its hosts like *T. cruciatum*, we do not think that *S. himalayana* will shift to Taiwan in the near future. First of all, *S. himalayana* is a root holoparasitic plant with limited distribution in southern China, such as Yunnan Province, and in some mountainous areas of neighboring countries, including India, Myanmar, Thailand, and Vietnam [41]. Secondly, according to the *Flora of Taiwan* and related literature, up to now, no occurrence record of *S. himalayana* has been reported in Taiwan [43,44]. Moreover, the Chinese mainland and Taiwan Province are separated by the Taiwan Strait, which has an average width of 200 km [45]. The fruit of *S. himalayana* is a spherical berry [25]. Although the dispersal mechanism of its fruit or seed is unclear, we think that it is unlikely for this parasite to spread and reach Taiwan in the coming decades.

Habitat overlap for *S. himalayana* with its hosts varies markedly, from the minimal overlap with *T. serrulatum* (0.04 × 10^4^ km^2^, 2.96%) to the complete overlap (100%) with both *T. obovatum* and *T. cruciatum*, matching the parasite’s entire current suitable area (Table S2). Niche overlap indices (*D* and *I*) also corroborate this variation. *T. serrulatum*, *T. planicaule*, and *T. obtectum* show only negligible overlap with *S. himalayana*, while *T. cruciatum* shows a moderate overlap tier, which is the highest overlap among all pairs (Table 5). Although future projections indicate an increase in the numerical overlap values for all pairs, the overall tiered structure remains largely unchanged. The only notable shift is for *S. himalayana* vs. *T. serrulatum*, which moves from negligible to low overlap (Table 5). This pattern may be attributed to the currently restricted distribution of *S. himalayana* in China, as its broader geographic range extends into other regions such as India, Myanmar, and Vietnam [41].

Notably, future projections show a decline for hosts with the largest current ranges (i.e., *T. obtectum* and *T. serrulatum*), but an expansion for those with smaller ranges (i.e., *T. planicaule*, *T. obovatum*, and *T. cruciatum*) (Table 4). This inverse relationship suggests that the responses of *S. himalayana* and its hosts to climate change may be partially mediated by the hosts’ extant distributional extents. One major reason for this is that while the suitable habitats of these hosts are primarily governed by climatic conditions, their sensitivity to climate change varies across species (see Table 3 and Figure 2, Figure 3, Figure 4, Figure 5 and Figure 6). Consequently, climate change may directly alter the future ranges of both the holoparasitic plant and its hosts, and indirectly shape the distribution of the endangered holoparasite *S. himalayana* by differentially modifying the available habitat of its various hosts. These shifts could also potentially lead to changes in the parasite’s host preference.

### 3.3. Implications for Sapria himalayana Conservation

This study is the first to identify key limiting factors and delineate suitable habitat range of *S. himalayana* in China, providing a reference for its future conservation. Firstly, we think that more conservation efforts should be paid to the suitable habitats of *S. himalayana*, particularly highly suitable areas. Considering that part of the predicted suitable habitat for *S. himalayana* in southern Yunnan is already located within the Xishuangbanna National Nature Reserve, southern China [38], while some other suitable areas lie just outside the boundary of the reserve, we propose considering enlarging the range of reserve appropriately or establishing a new plant conservation site to better protect wild *S. himalayana* populations. Notably, some areas in southeastern Tibet are expected to become suitable habitats for *S. himalayana* under several future scenarios in this study (e.g., SSP1-2.6 2070s, SSP1-2.6 2090s, SSP5-8.5 2090s) (Figure 1). Therefore, we recommend conducting supplementary field surveys and dynamic monitoring in these potential areas. Furthermore, *S. himalayana* is an obligate root holoparasite, forming an essential symbiotic association with multiple *Tetrastigma* host species [24,46]. Consequently, effective conservation strategies should simultaneously take into account the conservation of these host plants across the parasite’s distribution range and the conservation of *S. himalayana* itself as well.

In contrast to the overall expansion projected for *S. himalayana*, the five hosts exhibit divergent future habitat trends. In future scenarios, *T. obtectum* and *T. serrulatum* are expected to contract. While *T. planicaule*, *T. obovatum*, and *T. cruciatum* are projected to expand, the extent of increases is limited. *T. planicaule* and *T. obovatum* increase by only 2.49% and 0.95% on average, respectively. Although *T. cruciatum* shows a larger relative increase (8.26%), its average suitable area in the future (7.95 × 10^4^ km^2^) remains the smallest among all hosts (Table 4). Moreover, the future suitable areas for these three hosts fluctuate across climate scenarios, often asynchronously with *S. himalayana*. As exemplified by *T. obovatum*, its suitable area decreases under four scenarios (i.e., SSP1-2.6 2070s, SSP2-4.5 2050s, SSP2-4.5 2070s, SSP2-4.5 2090s) while *S. himalayana*’s increases, yet under SSP5-8.5 2050s, the trend reverses (Table 4). Furthermore, future habitat overlaps between *S. himalayana* and these hosts are generally projected to decline (e.g., from 68.69% to 67.20% with *T. planicaule*; from 100% to 89.78% with *T. obovatum*), or remain minimal despite an increase (e.g., from 2.96% to 8.06%, averaging only 0.15 × 10^4^ km^2^ with *T. cruciatum*) (Table S4). In summary, considering the divergent responses of multiple hosts, climate change will likely have adverse effects on *S. himalayana* in its distribution.

As an endophytic herb, *S. himalayana* is an obligate root holoparasite with high dependence on its hosts [23,24]. Furthermore, this parasitic plant is dioecious, and it has difficulties in pollination, resulting in low fruit set in the wild [36,47]. Our analysis indicates that an asynchrony exists in suitable habitat shift between *S. himalayana* and its hosts under varying climate scenarios. As a result, this mismatch could pose challenges to the future survival and distribution of *S. himalayana* in a warming climate.

### 3.4. The Limitations of This Study

Our study has certain limitations. Firstly, given that *S. himalayana* has few occurrence records, resulting from the geographically restricted distribution in China, we assessed its risk of niche truncation, although we did not employ a spatially nested hierarchical SDMs (n-SDMs) framework [48]. The results suggest that *S. himalayana* and its five hosts appear to have a high degree of niche truncation (Table 2), which indicate that the resulting projections may not reflect the entire ecological niche of the targeted species. They are better suited for “relative habitat suitability ranking” and “survey and conservation priority area screening” within China. Therefore, the key influencing factors identified in this study may only reflect the environmental situation of *S. himalayana* within China, and it is difficult to determine its ecological niches in other countries in Southeast Asia. Although developing n-SDMs contributes to improving model accuracy, we did not employ such a method because our research objective is to identify national-scale priority conservation areas and provide management recommendations for this endangered holoparasite in China.

Moreover, as a correlative model, MaxEnt prediction is based on known occurrence (or absence) points and associated environmental variables [49], which do not incorporate species-specific biological traits such as dispersal capability. In fact, although parasitic plants’ distribution is often constrained by their hosts, it is a great challenge to quantify host influence as a model variable due to the complexity of interspecific relationships [50,51]. In addition, this study did not take into account the preference of *S. himalayana* for different hosts.

## 4. Materials and Methods

### 4.1. Occurrence Records

The occurrence data of *Sapria himalayana* and its hosts were sourced through the following approaches. (1) Literature search: We used specific names, Latin names, and synonyms of each species as keywords to search for their occurrence records from *Flora of China*, provincial floras, relevant checklists, and academic papers. (2) Herbarium retrieval: We obtained specimen data with precise latitude–longitude coordinates or detailed location descriptions for each species from the Chinese Virtual Herbarium (CVH, https://www.cvh.ac.cn, accessed on 18 April 2025), National Specimen Information Infrastructure (NSII, http://www.nsii.org.cn, accessed on 18 April 2025), and Global Biodiversity Information Facility (GBIF, https://www.gbif.org/, accessed on 16 January 2026). (3) Image library: We searched for images of each species in the Plant Photo Bank of China (PPBC, http://ppbc.iplant.cn, accessed on 18 April 2025) and extracted corresponding detailed place names. (4) Field survey: We documented the spatial locations of these species’ wild populations via field investigations in eastern, southern, and southwestern China from 2023 to 2025.

**Figure 8:**
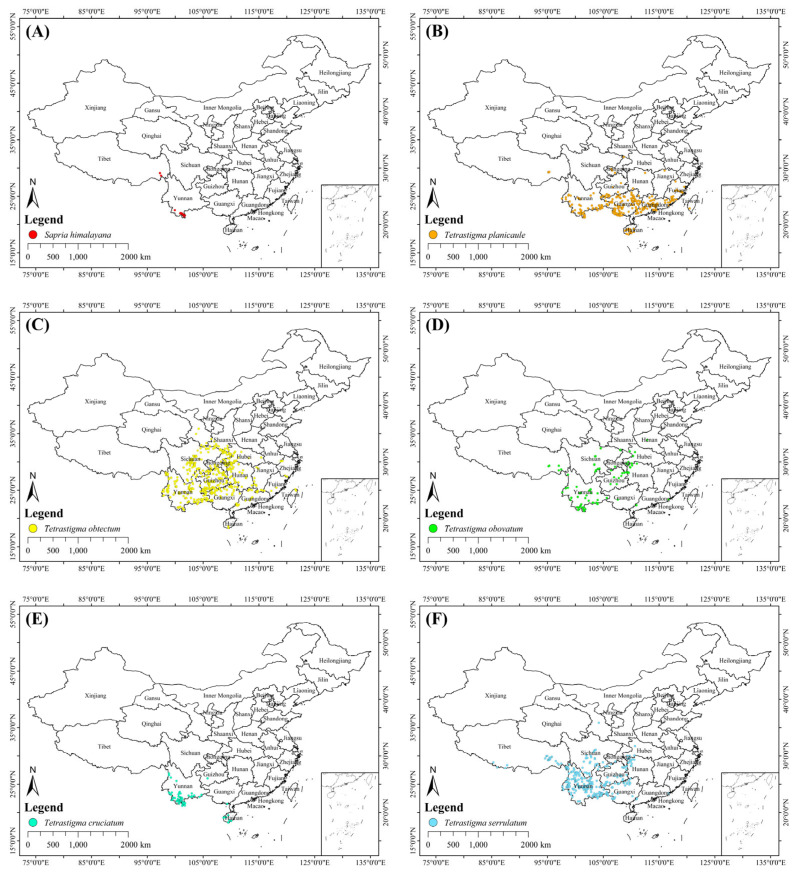
Occurrence records of *Sapria himalayana* and its five hosts in China. (**A**) *S. himalayana*; (**B**) *Tetrastigma planicaule*; (**C**) *T. obtectum*; (**D**) *T. obovatum*; (**E**) *T. cruciatum*; (**F**) *T. serrulatum*.

For records with specific location descriptions but lacking coordinate data, we used Google Earth (https://earth.google.com/web/, accessed on 26 April 2025) to obtain their latitude and longitude information, which was precise to two decimal places. Subsequently, we excluded duplicate records and those representing cultivated plants in schools, parks, and botanical gardens and so on. Then we spatially rarefied the occurrence data by using the Spatial Rare Occurrence Data for SDMs tool in SDMtoolbox v2.6 to ensure that only one occurrence point was retained per 1 km × 1 km grid cell [52]. Ultimately, we collected the coordinate information for each species with the following numbers: 22 for *S. himalayana*, 317 for *Tetrastigma planicaule*, 495 for *T. obtectum*, 93 for *T. obovatum*, 84 for *T. cruciatum*, and 329 for *T. serrulatum* (Table S3). The occurrence records for these six species are shown in Figure 8 and Table S3.

### 4.2. Environmental Variables

At regional scales, climate acts as a primary factor governing the geographic distribution of plants [53,54]. The six species spanned a three-step ladder of China in terms of occurrence records (Figure 8) and were mainly located in densely populated areas of southern China. Therefore, we selected topographic and anthropogenic variables besides climate. In total, 23 environmental variables were used as candidate predictors, which can be categorized into three groups: (1) Bioclimatic variables: We downloaded 19 bioclimatic variables from WorldClim, including both current and future climate data. Current bioclimatic data were based on WorldClim v2.1. Future climate data were derived from the Beijing Climate Center Climate System Model version 2 with Medium Resolution (BCC-CSM2-MR) in the Coupled Model Intercomparison Project Phase 6 (CMIP6). This model is considered suitable for Asia, particularly for China [40]. Because the scope of this research is in China, where the parasite and its multiple hosts occur, we only selected the BCC-CSM2-MR climate model in this study. We selected three Shared Socioeconomic Pathways (SSPs), namely SSP1-2.6 (optimistic scenario), SSP2-4.5 (moderate scenario), and SSP5-8.5 (pessimistic scenario), to represent different future climate change scenarios [55]. Each pathway contained three time periods (i.e., 2041–2060, 2061–2080, and 2081–2100). (2) Topographic variables: We downloaded elevation data from WorldClim (https://www.worldclim.org/, accessed on 3 May 2025) and extracted slope and aspect data from the Digital Elevation Model (DEM) (http://www.tuxingis.com, accessed on 3 May 2025). (3) Anthropogenic variables: We downloaded the global Human Influence (HI) dataset from the NASA Socioeconomic Data and Applications Center (SEDAC) (https://sedac.ciesin.columbia.edu, accessed on 3 May 2025) which comprised nine layers: population density, built-up areas, night-time light, land use/land cover, and human access features (roads, railways, coastlines, and navigable rivers) [56]. Detailed descriptions of each environmental variable are provided in Table S4.

To constrain modeling to the study area, we uniformly cropped all environmental variables to match China’s territory and resampled them to 30 arc-second spatial resolution. Subsequently, to avoid overfitting of models caused by multicollinearity among variables, we screened all 23 variables for each species (including *S. himalayana* and its five hosts) by using Spearman’s correlation coefficient. For two variables with an absolute coefficient value > 0.7, the one with the lower contribution was removed. If the value between two variables was ≤0.7, both were retained [57].

### 4.3. Model Construction

In this study, we once tried to use Biomod2 to construct an ensemble model for the targeted species. Unfortunately, we noted that the model’s performance was below expectations. The MaxEnt model is extensively employed due to its strong predictive performance, user-friendly operation, and ability to generate reliable projections at low sample sizes [58,59]. Existing studies have shown that for endangered plants, which often have limited distributions and few records, it seems a good choice to use the MaxEnt model [60]. Therefore, we used MaxEnt v3.4.4 to predict potential suitable habitats for *S. himalayana* and its five hosts under both current and future climate scenarios rather than ensemble models in Biomod2. Firstly, we used the ENMeval package in R v4.4.2 to select the optimal combination of RM and FC parameters for each model. Specifically, the RM value ranged from 0.5 to 4 (increments of 0.5) while the five FC features combined into six classes (i.e., L, H, LQ, LQH, LQHP, LQHPT; L = linear, Q = quadratic, H = hinge, P = product, T = threshold). The combination with ΔAICc = 0 was identified as the best modeling parameters [61,62]. During modeling, 75% of the occurrence data were selected as the training set and the remaining 25% as the testing set. To ensure projection accuracy, 10,000 background points were randomly generated and 10 bootstrap replicates were performed for each model. Meanwhile, we set corresponding RM and FC parameters while modeling for different species based on optimization results (Table 6). Additionally, we used a jackknife test to evaluate the contribution of each environmental variable to each species’ distribution.

We used the area under the receiver operating characteristic curve (AUC) and true skill statistic (TSS) to evaluate model performance [63]. The AUC value ranged from 0 to 1 and can be categorized into five levels: failing (0.5–0.6), poor (0.6–0.7), fair (0.7–0.8), good (0.8–0.9), and excellent (0.9–1.0) [64,65]. The TSS value ranged from –1 to 1, where 1 indicated perfect performance, while 0 or less suggested that the model performed no better than random prediction [3,66].

### 4.4. Geospatial Analysis

MaxEnt generated distribution predictions based on a logistic output format, which included a continuous habitat suitability index with values ranging from 0 (unsuitable) to 1 (perfectly suitable) [67]. We imported model outputs for *S. himalayana* and its five hosts into ArcGIS v10.8.1 in “.asc” format. Subsequently, we reclassified habitat suitability into four categories (i.e., highly suitable, moderately suitable, low suitable, and unsuitable) based on maximizing the sum of sensitivity and specificity (maxSSS) [68,69]. Given the considerable variation in maxSSS values among the six targeted species, we set a suitability threshold for each species (Table 7). Meanwhile, we used the Spatial Analyst tool to calculate overlapping areas of suitable habitat between *S. himalayana* and each of its hosts [19]. To reflect the shift in suitable habitats for *S. himalayana* and its five hosts under future climate scenarios, we used SDMtoolbox v2.6 in ArcGIS v10.8.1 to calculate the suitable distribution centroid under current and future scenarios for each species [70].

To evaluate niche truncation in *S. himalayana* and its five hosts, we calculated the niche unfilling index for each species using the “ecospat” package in R v4.4.2 [71,72]. Given that the core contribution of this study is to provide survey priorities and risk alerts within China, we did not further construct spatially nested hierarchical Species Distribution Models (n-SDMs). This approach can tackle the issue of species niche truncation through the integration of large-scale, low-resolution models with small-scale, high-resolution ones. However, the current methodological framework remains fragmented, having various nested strategies (e.g., “covariate”, “multiply”, etc.) [48,73], thus lacking a standardized, widely accepted methodology. Additionally, the multivariate environmental similarity surface (MESS) values for all six species were computed with the “dismo” package to assess extrapolation risks under future climate scenarios [74].

The niche overlap index quantified similarity in resource utilization and habitat preferences between different species [75]. To measure the niche overlap between *S. himalayana* and its five hosts across different climate scenarios, we selected Schoener’s *D* [76] and modified Hellinger distance (Hellinger’s *I*) [77]. These metrics were calculated in ENMTools v1.3.1.

Schoener’s *D* is defined as:

$$D\left(p_{X},\;p_{Y}\right)=1-\frac{1}{2}\sum_{i}|p_{X,i}-p_{Y,i}|$$

In which $p_{X,i}$ (or $p_{Y,i}$) is the normalized suitability score for species *X* (or *Y*) in grid cell *i*.

Schoener’s *D* quantified the similarity of potential distributions by comparing corresponding values in each grid cell. *D* value ranged from 0 to 1, where 0 indicated no niche overlap and 1 indicated identical potential distribution [71]. Following the method of [78], *D* value was classified into five levels: (1) extremely high overlap: 0.80–1.00; (2) high overlap: 0.60–0.80; (3) moderate overlap: 0.40–0.60; (4) low overlap: 0.20–0.40; (5) no or very limited overlap: 0.00–0.20.

Modified Hellinger’s *I* is defined as:

$$I\left(p_{X},\;p_{Y}\right)=1-\frac{1}{2}\left.\sqrt{\sum_{i}{\left(\sqrt{p_{X,i}}-\sqrt{p_{Y,i}}\right)}^{2}}\right.$$

*I* value ranged from 0 to 1, with a value of 0 indicating entirely divergent environmental requirements, and a value of 1 indicating that the environmental factor requirements for both species completely overlapped [79].

## 5. Conclusions

Our study delineates, for the first time, the suitable habitat range of the endangered endo-holoparasitic plant *Sapria himalayana* in China and identifies temperature-related climatic variables as key environmental factors influencing its distribution. Optimized MaxEnt modeling indicates that while the suitable habitat for *S. himalayana* will undergo a moderate increase under future climate scenarios, its multiple hosts show varying trends in potential habitat shifts. Together with the mismatch in suitability between the parasite and its hosts, divergent climatic sensitivities among host species could affect the survival and distribution of *S. himalayana*. Therefore, we recommend that future conservation efforts should prioritize: (1) supplementary field surveys of *S. himalayana* populations; (2) expansion and improved management of its protected areas; and (3) maintenance of its distinct ecological interactions with obligate hosts. In summary, this study provides an important reference for the conservation of *S. himalayana* in China, emphasizing the necessity of safeguarding its distinct hosts in the context of global warming.

## Figures and Tables

**Table 1:** Environmental extrapolation risk for *Sapria himalayana* and its five hosts, quantified by proportion (%) of pixels with multivariate environmental similarity surface (MESS) value < 0 under future climate scenarios.

Scenarios		Species					
		*Sapria himalayana*	*Tetrastigma planicaule*	*Tetrastigma obtectum*	*Tetrastigma obovatum*	*Tetrastigma cruciatum*	*Tetrastigma serrulatum*
SSP1-2.6	2050s	0.5967	0.5237	0.3308	0.3515	0.4334	0.4308
	2070s	1.4187	1.3862	1.2031	0.5172	0.4936	1.3672
	2090s	0.7044	0.6419	0.3827	0.6634	0.5590	0.7012
SSP2-4.5	2050s	0.7715	0.7019	0.5287	0.4845	0.5508	0.5164
	2070s	1.6132	1.5010	1.1757	0.8507	0.6090	1.6607
	2090s	2.1886	3.8092	0.5298	1.2102	1.7893	1.0131
SSP5-8.5	2050s	1.0201	0.9847	0.5971	1.1299	0.9029	1.2994
	2070s	2.6380	2.2541	1.5062	1.0826	1.2562	2.0254
	2090s	3.9101	11.4862	0.7580	1.4092	3.4301	1.5606

**Table 2:** Niche truncation assessment for *Sapria himalayana* and its five hosts.

Species	Niche Unfilling (%)	Global Occurrences (n)	China Occurrences (n)
*Sapria himalayana*	90.38	116	22
*Tetrastigma planicaule*	65.00	349	317
*Tetrastigma obtectum*	63.76	2710	495
*Tetrastigma obovatum*	18.18	93	76
*Tetrastigma cruciatum*	60.61	139	84
*Tetrastigma serrulatum*	37.65	577	329

Niche unfilling (%) means the proportion of occurrence points of species in China that are not covered within the global ecological niche space.

**Table 3:** Percent contribution of environmental factors of *Sapria himalayana* and its five hosts in China.

Factors	Percent Contribution (%)					
	*Sapria* *himalayana*	*Tetrastigma planicaule*	*Tetrastigma obtectum*	*Tetrastigma obovatum*	*Tetrastigma cruciatum*	*Tetrastigma serrulatum*
Bio1	**3.3**	-	-	-	1.4	-
Bio2	0.4	0.5	0.3	-	-	1.4
Bio3	**88.4**	-	-	**17.9**	**16.1**	-
Bio4	**4.8**	**5.7**	-	-	**72.9**	**33.7**
Bio5	-	-	-	-	-	-
Bio6	-	-	**19.9**	**51.4**	-	-
Bio7	-	-	2.4	-	1.6	-
Bio8	-	**1.6**	-	-	-	-
Bio9	-	-	-	-	-	2.3
Bio10	-	-	-	-	-	-
Bio11	-	**67.4**	-	-	-	-
Bio12	-	**16.2**	**40.1**	**7.8**	**3.2**	**9.3**
Bio13	0.5	-	-	-	**2.2**	-
Bio14	0	-	**15.2**	-	0	-
Bio15	-	-	-	1.2	-	1
Bio16	-	-	-	-	-	-
Bio17	0.2	1.5	-	**7.5**	-	**33.8**
Bio18	**1.2**	1.4	0.7	-	0.1	-
Bio19	-	-	-	-	0	-
Aspect	**0.6**	0.3	0.3	0.9	0.1	0.4
Elevation	-	0.6	**12.7**	1.5	-	**11.4**
Slope	0.4	0.5	0.7	0.5	0.1	0.6
HI	0.1	**4.4**	**7.8**	**11.2**	**2.2**	**6.1**

“-” represents the variables removed after collinear screening, and variables with the top five contribution rates are in bold.

**Table 4:** Dynamics of changes in the distribution area of *Sapria himalayana* and its five hosts under different climate scenarios.

Species	Scenarios		Low Suitable Areas		Moderately Suitable Areas		Highly Suitable Areas		Suitable Areas (Moderately and Highly)	
			Area (×10^4^ km^2^)	Trend (%)	Area (×10^4^ km^2^)	Trend (%)	Area (×10^4^ km^2^)	Trend (%)	Area (×10^4^ km^2^)	Trend (%)
*Sapria himalayana*	Current		2.28	-	0.69	-	0.66	-	1.35	-
	SSP1-2.6	2050s	2.87	↑25.88	0.85	↑23.19	0.99	↑50.00	1.84	↑36.30
		2070s	6.91	↑203.07	1.31	↑89.86	1.02	↑54.55	2.33	↑72.59
		2090s	4.25	↑86.40	1.19	↑72.46	1.00	↑51.52	2.19	↑62.22
		Average	4.68	↑105.26	1.12	↑62.32	1.00	↑51.52	2.12	↑57.04
	SSP2-4.5	2050s	4.86	↑113.16	1.50	↑117.39	0.96	↑45.45	2.46	↑82.22
		2070s	2.02	↓11.40	1.28	↑85.51	0.49	↓25.76	1.77	↑31.11
		2090s	2.82	↑23.68	1.40	↑102.90	0.65	↓1.52	2.05	↑51.85
		Average	3.23	↑41.67	1.39	↑101.45	0.70	↑6.06	2.09	↑54.81
	SSP5-8.5	2050s	4.35	↑90.79	0.82	↑18.84	0.47	↓28.79	1.29	↓4.44
		2070s	4.78	↑109.65	0.89	↑28.99	0.64	↓3.03	1.53	↑13.33
		2090s	3.57	↑56.58	0.88	↑27.54	0.46	↓30.30	1.34	↓0.74
		Average	4.23	↑85.53	0.86	↑24.64	0.52	↓21.21	1.38	↑2.22
	Total average		4.05	↑77.63	1.12	↑62.32	0.74	↑12.12	1.86	↑37.78
*Tetrastigma planicaule*	Current		33.66	-	31.05	-	5.63	-	36.68	-
	SSP1-2.6	2050s	31.15	↓7.46	34.82	↑12.14	4.88	↓13.32	39.70	↑8.23
		2070s	33.62	↓0.12	32.52	↑4.73	5.62	↓0.18	38.14	↑3.98
		2090s	38.92	↑15.63	29.64	↓4.54	5.99	↑6.39	35.63	↓2.86
		Average	34.56	↑2.67	32.33	↑4.12	5.50	↓2.31	37.82	↑3.11
	SSP2-4.5	2050s	31.35	↓6.86	33.89	↑9.15	5.68	↑0.89	39.57	↑7.88
		2070s	34.24	↑1.72	31.57	↑1.67	5.26	↓6.57	36.83	↑0.41
		2090s	38.08	↑13.13	30.63	↓1.35	5.44	↓3.37	36.07	↓1.66
		Average	34.56	↑2.67	32.03	↑3.16	5.46	↓3.02	37.49	↑2.21
	SSP5-8.5	2050s	35.11	↑4.31	31.17	↑0.39	6.40	↑13.68	37.57	↑2.43
		2070s	35.45	↑5.32	30.96	↓0.29	5.84	↑3.73	36.80	↑0.33
		2090s	34.02	↑1.07	32.62	↑5.06	5.46	↓3.02	38.08	↑3.82
		Average	34.86	↑3.57	31.58	↑1.71	5.90	↑4.80	37.48	↑2.18
	Total average		34.66	↑2.97	31.98	↑3.00	5.62	↓0.18	37.60	↑2.51
*Tetrastigma obtectum*	Current		76.08	-	63.07	-	8.63	-	71.70	-
	SSP1-2.6	2050s	76.13	↑0.07	62.18	↓1.41	8.82	↑2.20	71.00	↓0.98
		2070s	72.10	↓5.23	66.81	↑5.93	9.62	↑11.47	76.43	↑6.60
		2090s	81.69	↑7.37	59.77	↓5.23	9.35	↑8.34	69.12	↓3.60
		Average	76.64	↑0.74	62.92	↓0.24	9.26	↑7.30	72.18	↑0.67
	SSP2-4.5	2050s	79.03	↑3.88	60.82	↓3.57	9.37	↑8.57	70.19	↓2.11
		2070s	75.24	↓1.10	57.67	↓8.56	10.31	↑19.47	67.98	↓5.19
		2090s	73.62	↓3.23	57.42	↓8.96	8.82	↑2.20	66.24	↓7.62
		Average	75.96	↓0.16	58.64	↓7.02	9.50	↑10.08	68.14	↓4.97
	SSP5-8.5	2050s	78.61	↑3.33	61.26	↓2.87	9.49	↑9.97	70.75	↓1.32
		2070s	79.60	↑4.63	58.77	↓6.82	9.82	↑13.79	68.59	↓4.34
		2090s	76.55	↑0.62	53.99	↓14.40	9.95	↑15.30	63.94	↓10.82
		Average	78.25	↑2.85	58.01	↓8.02	9.75	↑12.98	67.76	↓5.50
	Total average		76.95	↑1.14	59.85	↓5.11	9.51	↑10.20	69.36	↓3.26
*Tetrastigma obovatum*	Current		74.85	-	20.82	-	9.60	-	30.42	-
	SSP1-2.6	2050s	62.22	↓16.87	20.97	↑0.72	9.69	↑0.94	30.66	↑0.79
		2070s	64.85	↓13.36	21.09	↑1.30	8.26	↓13.96	29.35	↓3.52
		2090s	61.87	↓17.34	26.19	↑25.79	9.80	↑2.08	35.99	↑18.31
		Average	62.98	↓15.86	22.75	↑9.27	9.25	↓3.65	32.00	↑5.19
	SSP2-4.5	2050s	69.71	↓6.87	21.95	↑5.43	8.14	↓15.21	30.09	↓1.08
		2070s	72.81	↓2.73	21.12	↑1.44	9.05	↓5.73	30.17	↓0.82
		2090s	59.70	↓20.24	17.56	↓15.66	7.70	↓19.79	25.26	↓16.96
		Average	67.41	↓9.94	20.21	↓2.93	8.30	↓13.54	28.51	↓6.28
	SSP5-8.5	2050s	75.95	↑1.47	23.87	↑14.65	11.55	↑20.31	35.42	↑16.44
		2070s	66.76	↓10.81	23.42	↑12.49	9.61	↑0.10	33.03	↑8.58
		2090s	67.43	↓9.91	18.80	↓9.70	7.66	↓20.21	26.46	↓13.02
		Average	70.05	↓6.41	22.03	↑5.81	9.61	↑0.10	31.64	↑4.01
	Total average		66.81	↓10.74	21.66	↑4.03	9.05	↓5.73	30.71	↑0.95
*Tetrastigma cruciatum*	Current		26.03	-	5.43	-	1.92	-	7.35	-
	SSP1-2.6	2050s	26.92	↑3.42	5.77	↑6.26	2.28	↑18.75	8.05	↑9.52
		2070s	28.74	↑10.41	7.11	↑30.94	2.63	↑36.98	9.74	↑32.52
		2090s	22.00	↓15.48	4.71	↓13.26	2.20	↑14.58	6.91	↓5.99
		Average	25.89	↓0.54	5.86	↑7.92	2.37	↑23.44	8.23	↑11.97
	SSP2-4.5	2050s	29.29	↑12.52	6.55	↑20.63	1.89	↓1.56	8.44	↑14.83
		2070s	21.33	↓18.06	4.90	↓9.76	2.32	↑20.83	7.22	↓1.77
		2090s	24.32	↓6.57	5.36	↓1.29	1.89	↓1.56	7.25	↓1.36
		Average	24.98	↓4.03	5.60	↑3.13	2.03	↑5.73	7.64	↑3.95
	SSP5-8.5	2050s	23.43	↓9.98	6.52	↑20.07	2.00	↑4.17	8.52	↑15.92
		2070s	26.60	↑2.19	5.10	↓6.08	2.18	↑13.54	7.28	↓0.95
		2090s	25.05	↓3.76	6.22	↑14.55	1.96	↑2.08	8.18	↑11.29
		Average	25.03	↓3.84	5.95	↑9.58	2.05	↑6.77	7.99	↑8.71
	Total average		25.30	↓2.80	5.80	↑6.81	2.15	↑11.98	7.95	↑8.16
*Tetrastigma serrulatum*	Current		64.84	-	27.70	-	10.39	-	38.09	-
	SSP1-2.6	2050s	62.11	↓4.21	29.50	↑6.50	9.68	↓6.83	39.18	↑2.86
		2070s	66.67	↑2.82	28.94	↑4.48	9.96	↓4.14	38.90	↑2.13
		2090s	65.13	↑0.45	26.55	↓4.15	9.46	↓8.95	36.01	↓5.46
		Average	64.64	↓0.31	28.33	↑2.27	9.70	↓6.64	38.03	↓0.16
	SSP2-4.5	2050s	61.37	↓5.35	27.95	↑0.90	9.32	↓10.30	37.27	↓2.15
		2070s	59.48	↓8.27	26.35	↓4.87	8.34	↓19.73	34.69	↓8.93
		2090s	68.23	↑5.23	28.27	↑2.06	8.52	↓18.00	36.79	↓3.41
		Average	63.03	↓2.79	27.52	↓0.65	8.73	↓15.98	36.25	↓4.83
	SSP5-8.5	2050s	68.43	↑5.54	25.37	↓8.41	8.96	↓13.76	34.33	↓9.87
		2070s	65.81	↑1.50	25.23	↓8.92	9.23	↓11.16	34.46	↓9.53
		2090s	68.67	↑5.91	26.87	↓3.00	9.71	↓6.54	36.58	↓3.96
		Average	67.64	↑4.32	25.82	↓6.79	9.30	↓10.49	35.12	↓7.80
	Total average		65.10	↑0.40	27.23	↓1.70	9.24	↓11.07	36.47	↓4.25

Up arrow (↑) means increase compared to the current; down arrow (↓) means decrease. Total average is the average of the suitable habitat areas under nine future climate scenarios. SSP = Shared Socioeconomic Pathways. SSP1-2.6 indicates optimistic pathways; SSP2-4.5 indicates moderate pathways; SSP5-8.5 indicates pessimistic pathways.

**Table 5:** Niche overlap measured by Schoener’s *D* and Hellinger’s *I* of *Sapria himalayana* and its five hosts under different climate scenarios.

Scenarios		Species									
		*Sapria himalayana* vs. *Tetrastigma planicaule*		*Sapria himalayana* vs. *Tetrastigma obtectum*		*Sapria himalayana* vs. *Tetrastigma obovatum*		*Sapria himalayana* vs. *Tetrastigma cruciatum*		*Sapria himalayana* vs. *Tetrastigma serrulatum*	
		*D*	*I*	*D*	*I*	*D*	*I*	*D*	*I*	*D*	*I*
Current		0.1299	0.5118	0.1435	0.4899	0.2162	0.5851	0.4413	0.7960	0.1903	0.5300
SSP1-2.6	2050s	↑0.2397	↑0.5903	↑0.2723	↑0.5839	↑0.3549	↑0.6768	↑0.5130	↑0.8183	↑0.3333	↑0.6370
	2070s	↑0.2379	↑0.5677	↑0.2440	↑0.5485	↑0.3462	↑0.6672	↑0.5921	↑0.8471	↑0.3105	↑0.6132
	2090s	↑0.2034	↑0.5441	↑0.2214	↑0.5312	↑0.3069	↑0.6258	↑0.5806	↑0.8507	↑0.2817	↑0.5992
	Average	↑0.2270	↑0.5674	↑0.2459	↑0.5545	↑0.3360	↑0.6566	↑0.5619	↑0.8387	↑0.3085	↑0.6165
SSP2-4.5	2050s	↑0.2064	↑0.5646	↑0.1979	↑0.5083	↑0.2938	↑0.6253	↑0.5987	↑0.8744	↑0.2648	↑0.5826
	2070s	↑0.2307	↑0.5878	↑0.2593	↑0.5909	↑0.3519	↑0.6905	↑0.5434	↑0.8528	↑0.3194	↑0.6449
	2090s	↑0.1671	↑0.5221	↑0.1759	↑0.5013	↑0.2747	↑0.6313	↑0.5283	↑0.8451	↑0.2333	↑0.5683
	Average	↑0.2014	↑0.5582	↑0.2110	↑0.5335	↑0.3068	↑0.6490	↑0.5568	↑0.8574	↑0.2725	↑0.5986
SSP5-8.5	2050s	↑0.1785	↑0.5711	↑0.2046	↑0.5065	↑0.2998	↑0.6469	↑0.5708	↑0.8545	↑0.2403	↑0.5650
	2070s	↑0.1755	↑0.5388	↓0.1423	↓0.4608	↑0.2440	↓0.5750	↑0.5612	↑0.8605	↑0.2190	↑0.5472
	2090s	↓0.0919	↓0.4574	↓0.0508	↓0.3522	↓0.1605	↓0.4999	↑0.5126	↑0.8444	↓0.1388	↓0.4640
	Average	↑0.1486	↑0.5224	↓0.1326	↓0.4398	↑0.2348	↓0.5739	↑0.5482	↑0.8531	↑0.1994	↓0.5254
Future average		↑0.1923	↑0.5493	↑0.1965	↑0.5093	↑0.2925	↑0.6265	↑0.5556	↑0.8498	↑0.2601	↑0.5802
Total average ± SD		↑0.1861 ± 0.0481	↑0.5456 ± 0.0406	↑0.1912 ± 0.0665	↑0.5074 ± 0.0681	↑0.2849 ± 0.0632	↑0.6224 ± 0.0567	↑0.5442 ± 0.0476	↑0.8444 ± 0.0221	↑0.2531 ± 0.0613	↑0.5751 ± 0.0538

Total average ± SD refers to the average value of Schoener’s *D* and Hellinger’s *I* of each pair under ten climate scenarios. Up arrow (↑) means increase compared to the current; down arrow (↓) means decrease.

**Table 6:** Optimization results of the MaxEnt model for *Sapria himalayana* and its five hosts.

Species	Feature Class	Regularization Multiplier
*Sapria himalayana*	LQHPT	1.5
*Tetrastigma planicaule*	LQHPT	1.5
*Tetrastigma obtectum*	LQH	1.5
*Tetrastigma obovatum*	LQ	0.5
*Tetrastigma cruciatum*	LQH	2.5
*Tetrastigma serrulatum*	LQHP	1.0

**Table 7:** Habitat suitability categorization based on maxSSS threshold values for *Sapria himalayana* and its five hosts.

Species	Unsuitable Area	Low Suitable Area	Moderately Suitable Area	Highly Suitable Area
*Sapria himalayana*	0–0.12	0.12–0.41	0.41–0.71	0.71–1.00
*Tetrastigma planicaule*	0–0.19	0.19–0.46	0.46–0.73	0.73–1.00
*Tetrastigma obtectum*	0–0.26	0.26–0.50	0.50–0.75	0.75–1.00
*Tetrastigma obovatum*	0–0.15	0.15–0.43	0.43–0.72	0.72–1.00
*Tetrastigma cruciatum*	0–0.05	0.05–0.37	0.37–0.68	0.68–1.00
*Tetrastigma serrulatum*	0–0.17	0.17–0.45	0.45–0.72	0.72–1.00

## Data Availability

The data presented in this study are available on request from the corresponding author.

## Supplementary Materials

The following supporting information can be downloaded at: https://www.mdpi.com/article/10.3390/plants15040574/s1, Table S1: The mean value (±SD) of the area under curve (AUC) and true skill statistic (TSS) of *Sapria himalayana* and its five hosts under different climate scenarios; Table S2: Dynamics of changes in overlap suitable areas of *Sapria himalayana* and its five hosts under different climate scenarios; Table S3: Occurrence records of *Sapria himalayana* and its five hosts in China; Table S4: Description of 23 candidate predictors used in MaxEnt model under different climate scenarios.
