# Supplementary material for: Assessing Potential Habitat Suitability of the Endangered Endo-Holoparasitic Sapria himalayana and Its Multiple Hosts in China Under Global Warming
Source: Plants (Basel). 2026 Feb 11;15(4):574. doi: 10.3390/plants15040574 (PMC12944323; doi:10.3390/plants15040574)
Supplement: Supplementary file 1 [file plants-15-00574-s001.zip › 20251225 Supplementary Tables.pdf]

Table S1: The mean value (±SD) of the area under curve (AUC) and true skill statistic (TSS) of *Sapria himalayana* and its five hosts under different climate scenarios.

**Scenarios**		**Species**											
		P: *Sapria himalayana*		H: *Tetrastigma planicaule*		H: *Tetrastigma obtectum*		H: *Tetrastigma obovatum*		H: *Tetrastigma cruciatum*		H: *Tetrastigma serrulatum*	
		**AUC**	**TSS**	**AUC**	**TSS**	**AUC**	**TSS**	**AUC**	**TSS**	**AUC**	**TSS**	**AUC**	**TSS**
Current		0.9969 ±0.0019	0.9421 ±0.0505	0.9727 ±0.0013	0.8908 ±0.0126	0.9431 ±0.0024	0.8080 ±0.0141	0.9648 ±0.0064	0.8212 ±0.0375	0.9918 ±0.0017	0.9152 ±0.0247	0.9636 ±0.0026	0.8498 ±0.0130
SSP1-2.6	2050s	0.9972 ±0.0015	0.9248 ±0.0489	0.9712 ±0.0011	0.8854 ±0.0078	0.9395 ±0.0033	0.7978 ±0.0113	0.9667 ±0.0041	0.8063 ±0.0301	0.9918 ±0.0014	0.9406 ±0.0100	0.9658 ±0.0016	0.8655 ±0.0126
	2070s	0.9974 ±0.0004	0.9218 ±0.0529	0.9725 ±0.0014	0.8923 ±0.0126	0.9401 ±0.0016	0.8057 ±0.0053	0.9689 ±0.0031	0.8266 ±0.0283	0.9914 ±0.0012	0.9310 ±0.0147	0.9633 ±0.0019	0.8535 ±0.0130
	2090s	0.9969 ±0.0009	0.9065 ±0.0357	0.9735 ±0.0016	0.8997 ±0.0117	0.9413 ±0.0017	0.7974 ±0.0068	0.9667 ±0.0039	0.8109 ±0.0186	0.9924 ±0.0020	0.9378 ±0.0167	0.9628 ±0.0019	0.8510 ±0.0066
SSP2-4.5	2050s	0.9972 ±0.0010	0.9205 ±0.0501	0.9734 ±0.0011	0.8976 ±0.0089	0.9416 ±0.0025	0.8039 ±0.0079	0.9683 ±0.0052	0.8151 ±0.0190	0.9922 ±0.0008	0.9394 ±0.0173	0.9650 ±0.0024	0.8515 ±0.0143
	2070s	0.9972 ±0.0016	0.9258 ±0.0610	0.9740 ±0.0016	0.8956 ±0.0115	0.9425 ±0.0016	0.7964 ±0.0102	0.9657 ±0.0020	0.8259 ±0.0330	0.9920 ±0.0016	0.9304 ±0.0217	0.9651 ±0.0011	0.8489 ±0.0131
	2090s	0.9979 ±0.0009	0.9187 ±0.0413	0.9725 ±0.0014	0.8982 ±0.0113	0.9416 ±0.0032	0.7968 ±0.0093	0.9697 ±0.0066	0.8098 ±0.0295	0.9920 ±0.0010	0.9390 ±0.0132	0.9622 ±0.0022	0.8474 ±0.0108
SSP5-8.5	2050s	0.9975	0.9350	0.9710	0.8805	0.9395	0.8043	0.9637	0.8042	0.9925	0.9430	0.9634	0.8499

	±0.0010	±0.0459	±0.0019	±0.0078	±0.0022	±0.0063	±0.0049	±0.0314	±0.0011	±0.0120	±0.0017	±0.0084
2070s	0.9983 ±0.0005	0.9567 ±0.0566	0.9720 ±0.0009	0.8899 ±0.0062	0.9397 ±0.0021	0.7997 ±0.0089	0.9665 ±0.0061	0.8161 ±0.0321	0.9916 ±0.0018	0.9312 ±0.0176	0.9644 ±0.0023	0.8454 ±0.0132
2090s	0.9981 ±0.0008	0.9586 ±0.0272	0.9733 ±0.0017	0.8906 ±0.0125	0.9402 ±0.0022	0.7961 ±0.0078	0.9667 ±0.0059	0.7926 ±0.0374	0.9915 ±0.0017	0.9286 ±0.0204	0.9635 ±0.0018	0.8470 ±0.0092

Table S2: Dynamics of changes in overlap suitable areas of *Sapria himalayana* and its five hosts under different climate scenarios.

Species	Scenarios		Overlap suitable areas		
			Area (× 10^4 km^2)	Overlap (%)	Trend (%)
Sapria himalayana vs *Tetrastigma planicaule*	Current		0.93	68.89	-
	SSP1-2.6	2050s	1.47	79.89	↑58.06
		2070s	1.53	65.67	↑64.52
		2090s	1.36	62.1	↑46.24
		Average	1.46	68.4	↑56.99
	SSP2-4.5	2050s	1.71	69.51	↑83.87
		2070s	1.01	57.06	↑8.60
		2090s	1.07	52.2	↑15.05
		Average	1.26	60.29	↑35.48
	SSP5-8.5	2050s	0.97	75.78	↑4.30
		2070s	1.11	72.55	↑19.35
		2090s	1.01	75.37	↑8.60
		Average	1.03	74.64	↑10.75
	Total average		1.25	67.2	↑34.41
Sapria himalayana vs *Tetrastigma obtectum*	Current		0.89	65.93	-
	SSP1-2.6	2050s	0.87	47.28	↓2.25
		2070s	1.43	61.37	↑60.67
		2090s	0.76	34.7	↓14.61
		Average	1.02	48.11	↑14.61
	SSP2-4.5	2050s	1.13	45.93	↑26.97
		2070s	0.56	31.64	↓37.08
		2090s	0.55	26.83	↓38.20
		Average	0.74	35.89	↓16.85
	SSP5-8.5	2050s	0.5	39.06	↓43.82
		2070s	0.3	19.61	↓66.29
		2090s	0.31	23.13	↓65.17
		Average	0.37	26.81	↓58.43
	Total average		0.71	38.17	↓20.22
Sapria himalayana vs *Tetrastigma obovatum*	Current		1.35	100	-
	SSP1-2.6	2050s	1.32	71.74	↓2.22
		2070s	1.96	84.12	↑45.19
		2090s	1.81	82.65	↑34.07
		Average	1.7	80.19	↑25.93
	SSP2-4.5	2050s	2.19	89.02	↑62.22
		2070s	1.77	100	↑31.11
		2090s	2.04	99.51	↑51.11

		Average	2	95.69	↑48.15
	SSP5-8.5	2050s	1.28	100	↓5.19
		2070s	1.42	92.81	↑5.19
		2090s	1.19	88.81	↓11.85
		Average	1.3	94.2	↓3.70
	Total average		1.66	89.78	↑22.96
Sapria himalayana vs *Tetrastigma cruciatum*	Current		1.35	100	-
	SSP1-2.6	2050s	1.83	99.46	↑35.56
		2070s	2.32	99.57	↑71.85
		2090s	2.17	99.09	↑60.74
		Average	2.1	99.53	↑55.56
	SSP2-4.5	2050s	2.45	99.59	↑81.48
		2070s	1.76	99.44	↑30.37
		2090s	2.04	99.51	↑51.11
		Average	2.08	99.52	↑54.07
	SSP5-8.5	2050s	1.28	100	↓5.19
		2070s	1.48	96.73	↑9.63
		2090s	1.26	94.03	↓6.67
		Average	1.34	97.1	↓0.74
	Total average		1.84	98.92	↑36.30
Sapria himalayana vs *Tetrastigma serrulatum*	Current		0.04	2.96	-
	SSP1-2.6	2050s	0.06	3.26	↑50.00
		2070s	0.18	7.73	↑350.00
		2090s	0.34	15.53	↑750.00
		Average	0.19	8.96	↑375.00
	SSP2-4.5	2050s	0.18	7.32	↑350.00
		2070s	0.07	3.95	↑75.00
		2090s	0.19	9.27	↑375.00
		Average	0.15	7.18	↑275.00
	SSP5-8.5	2050s	0.08	6.25	↑100.00
		2070s	0.13	8.5	↑225.00
		2090s	0.13	9.7	↑225.00
		Average	0.11	7.97	↑175.00
	Total average		0.15	8.06	↑275.00

Up arrow (↑) means increase compared to the current; down arrow (↓) means decrease. Total average is the average of the suitable habitat areas under nine future climate scenarios. SSP = Shared Socioeconomic Pathways. SSP1-2.6 indicates optimistic pathways; SSP2-4.5 indicates moderate pathways; SSP5-8.5 indicates pessimistic pathways.

Table S3: Occurrence records of *Sapria himalayana* and its five hosts in China.

No.	Species	Longitude（°）	Latitude（°）	No.	Species	Longitude（°）	Latitude（°）
1	*Sapria himalayana*	101.56	21.46	12	*Sapria himalayana*	101.20	21.87
2	*Sapria himalayana*	101.59	21.60	13	*Sapria himalayana*	101.34	21.87
3	*Sapria himalayana*	101.59	21.62	14	*Sapria himalayana*	101.20	21.88
4	*Sapria himalayana*	101.74	21.62	15	*Sapria himalayana*	101.25	21.90
5	*Sapria himalayana*	101.53	21.63	16	*Sapria himalayana*	101.27	21.93
6	*Sapria himalayana*	101.61	21.63	17	*Sapria himalayana*	101.10	21.99
7	*Sapria himalayana*	101.58	21.66	18	*Sapria himalayana*	100.89	21.99
8	*Sapria himalayana*	101.38	21.71	19	*Sapria himalayana*	101.00	22.04
9	*Sapria himalayana*	101.39	21.73	20	*Sapria himalayana*	99.93	22.56
10	*Sapria himalayana*	101.55	21.75	21	*Sapria himalayana*	97.47	28.66
11	*Sapria himalayana*	101.41	21.77	22	*Sapria himalayana*	97.26	29.11
1	*Tetrastigma planicaule*	95.17	29.28	160	*Tetrastigma planicaule*	108.79	24.97
2	*Tetrastigma planicaule*	95.18	29.24	161	*Tetrastigma planicaule*	108.84	24.91
3	*Tetrastigma planicaule*	95.21	29.26	162	*Tetrastigma planicaule*	108.85	18.96
4	*Tetrastigma planicaule*	95.33	29.33	163	*Tetrastigma planicaule*	109.17	18.36
5	*Tetrastigma planicaule*	97.58	24.63	164	*Tetrastigma planicaule*	109.18	19.11
6	*Tetrastigma planicaule*	98.29	24.81	165	*Tetrastigma planicaule*	109.21	19.08
7	*Tetrastigma planicaule*	98.30	24.43	166	*Tetrastigma planicaule*	109.41	24.28
8	*Tetrastigma planicaule*	98.74	25.13	167	*Tetrastigma planicaule*	109.45	19.23
9	*Tetrastigma planicaule*	98.80	25.30	168	*Tetrastigma planicaule*	109.51	18.25
10	*Tetrastigma*	98.81	25.29	169	*Tetrastigma*	109.52	19.13

No.	Species	Longitude（°）	Latitude（°）	No.	Species	Longitude（°）	Latitude（°）
	planicaule				*planicaule*		
11	*Tetrastigma planicaule*	98.96	24.09	170	*Tetrastigma planicaule*	109.55	22.27
12	*Tetrastigma planicaule*	99.08	23.30	171	*Tetrastigma planicaule*	109.70	18.62
13	*Tetrastigma planicaule*	99.10	23.30	172	*Tetrastigma planicaule*	109.70	18.63
14	*Tetrastigma planicaule*	99.16	25.12	173	*Tetrastigma planicaule*	109.77	19.20
15	*Tetrastigma planicaule*	99.40	23.55	174	*Tetrastigma planicaule*	109.83	19.03
16	*Tetrastigma planicaule*	99.46	22.25	175	*Tetrastigma planicaule*	109.96	23.50
17	*Tetrastigma planicaule*	99.58	22.33	176	*Tetrastigma planicaule*	109.98	18.79
18	*Tetrastigma planicaule*	99.93	22.56	177	*Tetrastigma planicaule*	109.99	24.63
19	*Tetrastigma planicaule*	100.43	24.76	178	*Tetrastigma planicaule*	110.02	23.54
20	*Tetrastigma planicaule*	100.56	22.05	179	*Tetrastigma planicaule*	110.03	18.52
21	*Tetrastigma planicaule*	100.70	23.50	180	*Tetrastigma planicaule*	110.03	18.50
22	*Tetrastigma planicaule*	100.71	24.62	181	*Tetrastigma planicaule*	110.04	18.51
23	*Tetrastigma planicaule*	100.80	22.01	182	*Tetrastigma planicaule*	110.05	25.26
24	*Tetrastigma planicaule*	100.83	24.45	183	*Tetrastigma planicaule*	110.06	25.69
25	*Tetrastigma planicaule*	100.86	22.70	184	*Tetrastigma planicaule*	110.07	25.53
26	*Tetrastigma planicaule*	100.90	22.09	185	*Tetrastigma planicaule*	110.12	24.26
27	*Tetrastigma planicaule*	100.92	21.85	186	*Tetrastigma planicaule*	110.13	24.26
28	*Tetrastigma planicaule*	100.97	23.33	187	*Tetrastigma planicaule*	110.18	24.13
29	*Tetrastigma planicaule*	100.98	22.18	188	*Tetrastigma planicaule*	110.20	22.91
30	*Tetrastigma planicaule*	101.00	22.36	189	*Tetrastigma planicaule*	110.21	24.67
31	*Tetrastigma planicaule*	101.06	22.50	190	*Tetrastigma planicaule*	110.24	24.18

No.	Species	Longitude（°）	Latitude（°）	No.	Species	Longitude（°）	Latitude（°）
32	*Tetrastigma planicaule*	101.23	21.99	191	*Tetrastigma planicaule*	110.28	23.94
33	*Tetrastigma planicaule*	101.24	21.98	192	*Tetrastigma planicaule*	110.29	24.73
34	*Tetrastigma planicaule*	101.25	21.94	193	*Tetrastigma planicaule*	110.30	25.08
35	*Tetrastigma planicaule*	101.28	21.92	194	*Tetrastigma planicaule*	110.31	25.06
36	*Tetrastigma planicaule*	101.50	22.17	195	*Tetrastigma planicaule*	110.32	24.03
37	*Tetrastigma planicaule*	101.52	21.28	196	*Tetrastigma planicaule*	110.32	26.39
38	*Tetrastigma planicaule*	101.56	21.46	197	*Tetrastigma planicaule*	110.35	25.17
39	*Tetrastigma planicaule*	101.57	21.48	198	*Tetrastigma planicaule*	110.45	24.85
40	*Tetrastigma planicaule*	101.59	21.60	199	*Tetrastigma planicaule*	110.46	19.25
41	*Tetrastigma planicaule*	101.64	21.73	200	*Tetrastigma planicaule*	110.46	23.52
42	*Tetrastigma planicaule*	101.66	21.48	201	*Tetrastigma planicaule*	110.48	18.68
43	*Tetrastigma planicaule*	101.67	21.27	202	*Tetrastigma planicaule*	110.52	24.19
44	*Tetrastigma planicaule*	101.68	21.62	203	*Tetrastigma planicaule*	110.53	24.92
45	*Tetrastigma planicaule*	101.70	21.28	204	*Tetrastigma planicaule*	110.57	24.13
46	*Tetrastigma planicaule*	102.26	22.88	205	*Tetrastigma planicaule*	110.58	24.61
47	*Tetrastigma planicaule*	102.74	25.14	206	*Tetrastigma planicaule*	110.62	24.49
48	*Tetrastigma planicaule*	102.75	25.12	207	*Tetrastigma planicaule*	110.70	24.48
49	*Tetrastigma planicaule*	102.96	23.38	208	*Tetrastigma planicaule*	110.80	24.17
50	*Tetrastigma planicaule*	103.09	22.79	209	*Tetrastigma planicaule*	110.82	24.31
51	*Tetrastigma planicaule*	103.09	22.80	210	*Tetrastigma planicaule*	110.83	23.10
52	*Tetrastigma planicaule*	103.15	22.92	211	*Tetrastigma planicaule*	110.85	21.92
53	*Tetrastigma*	103.26	25.56	212	*Tetrastigma*	110.93	22.82

No.	Species	Longitude（°）	Latitude（°）	No.	Species	Longitude（°）	Latitude（°）
	planicaule				*planicaule*		
54	*Tetrastigma planicaule*	103.68	22.98	213	*Tetrastigma planicaule*	110.95	22.35
55	*Tetrastigma planicaule*	103.69	22.98	214	*Tetrastigma planicaule*	110.99	24.10
56	*Tetrastigma planicaule*	103.82	22.90	215	*Tetrastigma planicaule*	111.02	23.73
57	*Tetrastigma planicaule*	103.94	22.53	216	*Tetrastigma planicaule*	111.03	22.45
58	*Tetrastigma planicaule*	103.97	22.52	217	*Tetrastigma planicaule*	111.20	22.02
59	*Tetrastigma planicaule*	104.23	23.39	218	*Tetrastigma planicaule*	111.21	23.70
60	*Tetrastigma planicaule*	104.25	23.37	219	*Tetrastigma planicaule*	111.25	22.08
61	*Tetrastigma planicaule*	104.39	23.01	220	*Tetrastigma planicaule*	111.30	23.49
62	*Tetrastigma planicaule*	104.40	23.02	221	*Tetrastigma planicaule*	111.31	24.23
63	*Tetrastigma planicaule*	104.44	22.75	222	*Tetrastigma planicaule*	111.34	25.09
64	*Tetrastigma planicaule*	104.55	23.03	223	*Tetrastigma planicaule*	111.57	22.77
65	*Tetrastigma planicaule*	104.58	24.86	224	*Tetrastigma planicaule*	111.61	23.84
66	*Tetrastigma planicaule*	104.67	23.44	225	*Tetrastigma planicaule*	111.62	24.10
67	*Tetrastigma planicaule*	104.68	23.04	226	*Tetrastigma planicaule*	111.72	22.25
68	*Tetrastigma planicaule*	104.70	23.13	227	*Tetrastigma planicaule*	111.78	22.18
69	*Tetrastigma planicaule*	104.74	22.97	228	*Tetrastigma planicaule*	112.01	22.56
70	*Tetrastigma planicaule*	104.79	23.38	229	*Tetrastigma planicaule*	112.04	22.93
71	*Tetrastigma planicaule*	104.82	23.18	230	*Tetrastigma planicaule*	112.18	23.92
72	*Tetrastigma planicaule*	104.84	22.94	231	*Tetrastigma planicaule*	112.19	23.93
73	*Tetrastigma planicaule*	104.90	25.08	232	*Tetrastigma planicaule*	112.20	23.93
74	*Tetrastigma planicaule*	104.94	25.10	233	*Tetrastigma planicaule*	112.22	22.69

No.	Species	Longitude（°）	Latitude（°）	No.	Species	Longitude（°）	Latitude（°）
75	*Tetrastigma planicaule*	105.33	24.77	234	*Tetrastigma planicaule*	112.23	22.70
76	*Tetrastigma planicaule*	105.39	25.13	235	*Tetrastigma planicaule*	112.34	23.09
77	*Tetrastigma planicaule*	105.45	24.19	236	*Tetrastigma planicaule*	112.41	22.86
78	*Tetrastigma planicaule*	105.62	23.63	237	*Tetrastigma planicaule*	112.45	23.03
79	*Tetrastigma planicaule*	105.63	23.63	238	*Tetrastigma planicaule*	112.47	23.05
80	*Tetrastigma planicaule*	105.64	25.39	239	*Tetrastigma planicaule*	112.48	23.08
81	*Tetrastigma planicaule*	105.81	24.98	240	*Tetrastigma planicaule*	112.50	29.15
82	*Tetrastigma planicaule*	105.83	23.42	241	*Tetrastigma planicaule*	112.53	23.16
83	*Tetrastigma planicaule*	105.96	25.26	242	*Tetrastigma planicaule*	112.54	23.17
84	*Tetrastigma planicaule*	106.02	25.16	243	*Tetrastigma planicaule*	112.57	23.17
85	*Tetrastigma planicaule*	106.04	25.10	244	*Tetrastigma planicaule*	112.87	22.83
86	*Tetrastigma planicaule*	106.08	23.88	245	*Tetrastigma planicaule*	112.99	23.74
87	*Tetrastigma planicaule*	106.12	25.05	246	*Tetrastigma planicaule*	113.05	23.69
88	*Tetrastigma planicaule*	106.30	23.99	247	*Tetrastigma planicaule*	113.22	23.57
89	*Tetrastigma planicaule*	106.40	29.84	248	*Tetrastigma planicaule*	113.27	24.78
90	*Tetrastigma planicaule*	106.46	23.91	249	*Tetrastigma planicaule*	113.34	23.12
91	*Tetrastigma planicaule*	106.55	29.57	250	*Tetrastigma planicaule*	113.36	23.12
92	*Tetrastigma planicaule*	106.57	24.12	251	*Tetrastigma planicaule*	113.37	23.18
93	*Tetrastigma planicaule*	106.60	29.55	252	*Tetrastigma planicaule*	113.40	23.06
94	*Tetrastigma planicaule*	106.62	23.90	253	*Tetrastigma planicaule*	113.42	24.19
95	*Tetrastigma planicaule*	106.66	24.50	254	*Tetrastigma planicaule*	113.59	23.55
96	*Tetrastigma*	106.69	26.58	255	*Tetrastigma*	113.67	24.27

No.	Species	Longitude（°）	Latitude（°）	No.	Species	Longitude（°）	Latitude（°）
	planicaule				*planicaule*		
97	*Tetrastigma planicaule*	106.70	22.30	256	*Tetrastigma planicaule*	113.74	25.02
98	*Tetrastigma planicaule*	106.75	22.12	257	*Tetrastigma planicaule*	113.81	23.74
99	*Tetrastigma planicaule*	106.79	22.11	258	*Tetrastigma planicaule*	113.86	23.70
100	*Tetrastigma planicaule*	106.80	22.31	259	*Tetrastigma planicaule*	113.88	23.69
101	*Tetrastigma planicaule*	106.82	22.39	260	*Tetrastigma planicaule*	113.89	23.64
102	*Tetrastigma planicaule*	106.86	22.06	261	*Tetrastigma planicaule*	113.92	22.63
103	*Tetrastigma planicaule*	106.94	22.86	262	*Tetrastigma planicaule*	113.95	23.81
104	*Tetrastigma planicaule*	107.02	21.86	263	*Tetrastigma planicaule*	113.98	22.53
105	*Tetrastigma planicaule*	107.03	22.44	264	*Tetrastigma planicaule*	113.99	24.40
106	*Tetrastigma planicaule*	107.06	22.24	265	*Tetrastigma planicaule*	114.02	23.29
107	*Tetrastigma planicaule*	107.07	22.13	266	*Tetrastigma planicaule*	114.06	22.55
108	*Tetrastigma planicaule*	107.10	29.16	267	*Tetrastigma planicaule*	114.13	24.35
109	*Tetrastigma planicaule*	107.11	22.44	268	*Tetrastigma planicaule*	114.18	22.58
110	*Tetrastigma planicaule*	107.11	22.76	269	*Tetrastigma planicaule*	114.21	22.58
111	*Tetrastigma planicaule*	107.14	23.44	270	*Tetrastigma planicaule*	114.25	23.50
112	*Tetrastigma planicaule*	107.20	22.83	271	*Tetrastigma planicaule*	114.26	23.37
113	*Tetrastigma planicaule*	107.33	22.01	272	*Tetrastigma planicaule*	114.27	23.38
114	*Tetrastigma planicaule*	107.35	24.84	273	*Tetrastigma planicaule*	114.28	23.18
115	*Tetrastigma planicaule*	107.45	21.68	274	*Tetrastigma planicaule*	114.35	22.69
116	*Tetrastigma planicaule*	107.47	24.92	275	*Tetrastigma planicaule*	114.40	23.31
117	*Tetrastigma planicaule*	107.57	21.92	276	*Tetrastigma planicaule*	114.42	23.06

No.	Species	Longitude（°）	Latitude（°）	No.	Species	Longitude（°）	Latitude（°）
118	*Tetrastigma planicaule*	107.58	21.72	277	*Tetrastigma planicaule*	114.49	22.61
119	*Tetrastigma planicaule*	107.58	23.33	278	*Tetrastigma planicaule*	114.51	23.59
120	*Tetrastigma planicaule*	107.60	25.05	279	*Tetrastigma planicaule*	114.54	22.52
121	*Tetrastigma planicaule*	107.62	21.62	280	*Tetrastigma planicaule*	114.69	23.74
122	*Tetrastigma planicaule*	107.63	25.75	281	*Tetrastigma planicaule*	114.70	23.74
123	*Tetrastigma planicaule*	107.67	22.86	282	*Tetrastigma planicaule*	114.71	23.47
124	*Tetrastigma planicaule*	107.68	23.18	283	*Tetrastigma planicaule*	114.75	23.50
125	*Tetrastigma planicaule*	107.70	23.17	284	*Tetrastigma planicaule*	115.99	29.54
126	*Tetrastigma planicaule*	107.75	22.93	285	*Tetrastigma planicaule*	116.05	24.28
127	*Tetrastigma planicaule*	107.76	22.94	286	*Tetrastigma planicaule*	116.10	23.85
128	*Tetrastigma planicaule*	107.83	22.46	287	*Tetrastigma planicaule*	116.40	24.40
129	*Tetrastigma planicaule*	107.84	22.46	288	*Tetrastigma planicaule*	116.61	23.91
130	*Tetrastigma planicaule*	107.86	21.85	289	*Tetrastigma planicaule*	116.70	23.37
131	*Tetrastigma planicaule*	107.87	22.42	290	*Tetrastigma planicaule*	116.94	23.87
132	*Tetrastigma planicaule*	107.89	23.26	291	*Tetrastigma planicaule*	117.12	24.12
133	*Tetrastigma planicaule*	107.90	21.85	292	*Tetrastigma planicaule*	117.21	24.88
134	*Tetrastigma planicaule*	107.92	24.83	293	*Tetrastigma planicaule*	117.22	24.56
135	*Tetrastigma planicaule*	107.94	25.32	294	*Tetrastigma planicaule*	117.23	24.88
136	*Tetrastigma planicaule*	107.95	21.81	295	*Tetrastigma planicaule*	117.30	24.37
137	*Tetrastigma planicaule*	107.96	22.10	296	*Tetrastigma planicaule*	117.31	24.70
138	*Tetrastigma planicaule*	107.97	21.53	297	*Tetrastigma planicaule*	117.31	24.71
139	*Tetrastigma*	107.98	22.15	298	*Tetrastigma*	117.36	24.28

No.	Species	Longitude（°）	Latitude（°）	No.	Species	Longitude（°）	Latitude（°）
	planicaule				*planicaule*		
140	*Tetrastigma planicaule*	108.00	21.74	299	*Tetrastigma planicaule*	117.37	24.52
141	*Tetrastigma planicaule*	108.05	21.61	300	*Tetrastigma planicaule*	117.51	24.42
142	*Tetrastigma planicaule*	108.09	24.69	301	*Tetrastigma planicaule*	117.52	25.00
143	*Tetrastigma planicaule*	108.10	23.93	302	*Tetrastigma planicaule*	117.59	24.39
144	*Tetrastigma planicaule*	108.16	27.34	303	*Tetrastigma planicaule*	117.63	24.22
145	*Tetrastigma planicaule*	108.20	23.04	304	*Tetrastigma planicaule*	117.66	24.49
146	*Tetrastigma planicaule*	108.22	22.97	305	*Tetrastigma planicaule*	117.92	25.95
147	*Tetrastigma planicaule*	108.25	22.10	306	*Tetrastigma planicaule*	117.95	27.64
148	*Tetrastigma planicaule*	108.31	22.83	307	*Tetrastigma planicaule*	118.08	24.45
149	*Tetrastigma planicaule*	108.35	21.62	308	*Tetrastigma planicaule*	118.10	24.45
150	*Tetrastigma planicaule*	108.37	22.84	309	*Tetrastigma planicaule*	118.19	26.17
151	*Tetrastigma planicaule*	108.38	22.79	310	*Tetrastigma planicaule*	118.38	25.95
152	*Tetrastigma planicaule*	108.40	24.19	311	*Tetrastigma planicaule*	118.80	25.86
153	*Tetrastigma planicaule*	108.52	23.06	312	*Tetrastigma planicaule*	118.93	25.87
154	*Tetrastigma planicaule*	108.60	23.43	313	*Tetrastigma planicaule*	118.96	25.78
155	*Tetrastigma planicaule*	108.62	23.50	314	*Tetrastigma planicaule*	119.09	25.88
156	*Tetrastigma planicaule*	108.65	21.98	315	*Tetrastigma planicaule*	119.22	25.67
157	*Tetrastigma planicaule*	108.66	31.95	316	*Tetrastigma planicaule*	119.51	26.68
158	*Tetrastigma planicaule*	108.67	24.98	317	*Tetrastigma planicaule*	120.47	22.87
159	*Tetrastigma planicaule*	108.68	24.97				
1	*Tetrastigma obtectum*	97.70	24.75	249	*Tetrastigma obtectum*	106.14	26.25

No.	Species	Longitude（°）	Latitude（°）	No.	Species	Longitude（°）	Latitude（°）
2	*Tetrastigma obtectum*	97.85	24.02	250	*Tetrastigma obtectum*	106.17	25.25
3	*Tetrastigma obtectum*	97.93	24.72	251	*Tetrastigma obtectum*	106.18	32.75
4	*Tetrastigma obtectum*	97.94	25.10	252	*Tetrastigma obtectum*	106.23	32.77
5	*Tetrastigma obtectum*	97.99	24.09	253	*Tetrastigma obtectum*	106.24	28.31
6	*Tetrastigma obtectum*	98.31	28.13	254	*Tetrastigma obtectum*	106.26	29.29
7	*Tetrastigma obtectum*	98.32	28.00	255	*Tetrastigma obtectum*	106.29	23.16
8	*Tetrastigma obtectum*	98.33	28.08	256	*Tetrastigma obtectum*	106.35	24.45
9	*Tetrastigma obtectum*	98.34	27.87	257	*Tetrastigma obtectum*	106.36	24.47
10	*Tetrastigma obtectum*	98.35	27.73	258	*Tetrastigma obtectum*	106.38	24.50
11	*Tetrastigma obtectum*	98.50	25.03	259	*Tetrastigma obtectum*	106.39	28.52
12	*Tetrastigma obtectum*	98.52	24.78	260	*Tetrastigma obtectum*	106.40	25.27
13	*Tetrastigma obtectum*	98.54	27.71	261	*Tetrastigma obtectum*	106.41	28.50
14	*Tetrastigma obtectum*	98.57	27.70	262	*Tetrastigma obtectum*	106.42	23.13
15	*Tetrastigma obtectum*	98.58	25.21	263	*Tetrastigma obtectum*	106.45	28.90
16	*Tetrastigma obtectum*	98.66	27.91	264	*Tetrastigma obtectum*	106.46	24.60
17	*Tetrastigma obtectum*	98.67	27.74	265	*Tetrastigma obtectum*	106.47	26.56
18	*Tetrastigma obtectum*	98.69	24.59	266	*Tetrastigma obtectum*	106.48	34.05
19	*Tetrastigma obtectum*	98.70	25.74	267	*Tetrastigma obtectum*	106.52	24.83
20	*Tetrastigma obtectum*	98.75	25.67	268	*Tetrastigma obtectum*	106.53	24.51
21	*Tetrastigma obtectum*	98.77	24.83	269	*Tetrastigma obtectum*	106.55	24.78
22	*Tetrastigma obtectum*	98.79	25.32	270	*Tetrastigma obtectum*	106.56	24.34
23	*Tetrastigma*	98.85	26.91	271	*Tetrastigma*	106.57	24.35

No.	Species	Longitude（°）	Latitude（°）	No.	Species	Longitude（°）	Latitude（°）
	obtectum				*obtectum*		
24	*Tetrastigma obtectum*	98.86	25.84	272	*Tetrastigma obtectum*	106.62	23.30
25	*Tetrastigma obtectum*	98.87	27.10	273	*Tetrastigma obtectum*	106.63	23.25
26	*Tetrastigma obtectum*	98.88	27.13	274	*Tetrastigma obtectum*	106.65	23.13
27	*Tetrastigma obtectum*	99.12	23.75	275	*Tetrastigma obtectum*	106.66	25.51
28	*Tetrastigma obtectum*	99.28	27.18	276	*Tetrastigma obtectum*	106.67	26.44
29	*Tetrastigma obtectum*	99.29	27.18	277	*Tetrastigma obtectum*	106.68	26.59
30	*Tetrastigma obtectum*	99.71	24.21	278	*Tetrastigma obtectum*	106.69	26.58
31	*Tetrastigma obtectum*	99.72	24.22	279	*Tetrastigma obtectum*	106.75	25.43
32	*Tetrastigma obtectum*	99.90	26.53	280	*Tetrastigma obtectum*	106.76	25.54
33	*Tetrastigma obtectum*	99.93	24.58	281	*Tetrastigma obtectum*	106.79	24.54
34	*Tetrastigma obtectum*	99.95	26.11	282	*Tetrastigma obtectum*	106.82	27.87
35	*Tetrastigma obtectum*	100.04	25.64	283	*Tetrastigma obtectum*	106.83	32.35
36	*Tetrastigma obtectum*	100.14	25.65	284	*Tetrastigma obtectum*	106.88	25.32
37	*Tetrastigma obtectum*	100.16	25.69	285	*Tetrastigma obtectum*	106.93	26.55
38	*Tetrastigma obtectum*	100.18	26.56	286	*Tetrastigma obtectum*	106.97	26.45
39	*Tetrastigma obtectum*	100.22	25.58	287	*Tetrastigma obtectum*	107.00	33.62
40	*Tetrastigma obtectum*	100.23	25.60	288	*Tetrastigma obtectum*	107.03	32.84
41	*Tetrastigma obtectum*	100.36	26.78	289	*Tetrastigma obtectum*	107.04	24.54
42	*Tetrastigma obtectum*	100.38	27.28	290	*Tetrastigma obtectum*	107.07	29.51
43	*Tetrastigma obtectum*	100.45	21.96	291	*Tetrastigma obtectum*	107.10	29.16
44	*Tetrastigma obtectum*	100.49	21.99	292	*Tetrastigma obtectum*	107.11	25.46

No.	Species	Longitude（°）	Latitude（°）	No.	Species	Longitude（°）	Latitude（°）
45	*Tetrastigma obtectum*	100.57	21.86	293	*Tetrastigma obtectum*	107.13	28.58
46	*Tetrastigma obtectum*	100.64	24.40	294	*Tetrastigma obtectum*	107.14	32.51
47	*Tetrastigma obtectum*	100.71	24.62	295	*Tetrastigma obtectum*	107.15	24.79
48	*Tetrastigma obtectum*	100.76	24.36	296	*Tetrastigma obtectum*	107.17	25.00
49	*Tetrastigma obtectum*	100.80	22.01	297	*Tetrastigma obtectum*	107.20	24.16
50	*Tetrastigma obtectum*	100.83	24.45	298	*Tetrastigma obtectum*	107.21	24.16
51	*Tetrastigma obtectum*	100.87	24.44	299	*Tetrastigma obtectum*	107.32	28.83
52	*Tetrastigma obtectum*	100.90	22.09	300	*Tetrastigma obtectum*	107.33	33.15
53	*Tetrastigma obtectum*	100.98	22.78	301	*Tetrastigma obtectum*	107.37	28.95
54	*Tetrastigma obtectum*	101.32	21.87	302	*Tetrastigma obtectum*	107.42	27.74
55	*Tetrastigma obtectum*	101.42	21.68	303	*Tetrastigma obtectum*	107.45	32.84
56	*Tetrastigma obtectum*	101.50	23.94	304	*Tetrastigma obtectum*	107.48	27.77
57	*Tetrastigma obtectum*	101.50	29.00	305	*Tetrastigma obtectum*	107.51	25.10
58	*Tetrastigma obtectum*	101.51	29.00	306	*Tetrastigma obtectum*	107.53	24.98
59	*Tetrastigma obtectum*	101.57	21.48	307	*Tetrastigma obtectum*	107.55	25.82
60	*Tetrastigma obtectum*	101.63	24.70	308	*Tetrastigma obtectum*	107.62	29.35
61	*Tetrastigma obtectum*	101.64	24.69	309	*Tetrastigma obtectum*	107.68	28.74
62	*Tetrastigma obtectum*	101.67	26.07	310	*Tetrastigma obtectum*	107.71	28.37
63	*Tetrastigma obtectum*	101.80	23.57	311	*Tetrastigma obtectum*	107.75	25.89
64	*Tetrastigma obtectum*	101.81	24.57	312	*Tetrastigma obtectum*	107.78	27.12
65	*Tetrastigma obtectum*	101.82	23.57	313	*Tetrastigma obtectum*	107.79	32.74
66	*Tetrastigma*	102.02	23.61	314	*Tetrastigma*	107.88	25.42

No.	Species	Longitude（°）	Latitude（°）	No.	Species	Longitude（°）	Latitude（°）
	obtectum				*obtectum*		
67	*Tetrastigma obtectum*	102.07	29.60	315	*Tetrastigma obtectum*	107.89	28.09
68	*Tetrastigma obtectum*	102.11	26.89	316	*Tetrastigma obtectum*	107.90	26.90
69	*Tetrastigma obtectum*	102.12	26.88	317	*Tetrastigma obtectum*	107.94	25.32
70	*Tetrastigma obtectum*	102.17	28.55	318	*Tetrastigma obtectum*	107.95	28.06
71	*Tetrastigma obtectum*	102.18	28.55	319	*Tetrastigma obtectum*	108.03	25.13
72	*Tetrastigma obtectum*	102.21	22.82	320	*Tetrastigma obtectum*	108.04	32.06
73	*Tetrastigma obtectum*	102.23	29.91	321	*Tetrastigma obtectum*	108.06	32.22
74	*Tetrastigma obtectum*	102.24	24.27	322	*Tetrastigma obtectum*	108.08	26.22
75	*Tetrastigma obtectum*	102.25	26.67	323	*Tetrastigma obtectum*	108.10	23.93
76	*Tetrastigma obtectum*	102.27	27.90	324	*Tetrastigma obtectum*	108.11	27.13
77	*Tetrastigma obtectum*	102.39	22.99	325	*Tetrastigma obtectum*	108.12	27.03
78	*Tetrastigma obtectum*	102.40	23.00	326	*Tetrastigma obtectum*	108.19	30.02
79	*Tetrastigma obtectum*	102.41	25.54	327	*Tetrastigma obtectum*	108.23	23.64
80	*Tetrastigma obtectum*	102.43	25.22	328	*Tetrastigma obtectum*	108.25	24.83
81	*Tetrastigma obtectum*	102.45	22.99	329	*Tetrastigma obtectum*	108.29	32.13
82	*Tetrastigma obtectum*	102.46	22.98	330	*Tetrastigma obtectum*	108.40	28.00
83	*Tetrastigma obtectum*	102.47	25.55	331	*Tetrastigma obtectum*	108.41	28.00
84	*Tetrastigma obtectum*	102.50	25.22	332	*Tetrastigma obtectum*	108.42	27.05
85	*Tetrastigma obtectum*	102.53	29.77	333	*Tetrastigma obtectum*	108.45	31.82
86	*Tetrastigma obtectum*	102.57	28.74	334	*Tetrastigma obtectum*	108.49	31.52
87	*Tetrastigma obtectum*	102.58	25.37	335	*Tetrastigma obtectum*	108.51	27.64

No.	Species	Longitude（°）	Latitude（°）	No.	Species	Longitude（°）	Latitude（°）
88	*Tetrastigma obtectum*	102.59	25.87	336	*Tetrastigma obtectum*	108.57	31.97
89	*Tetrastigma obtectum*	102.60	30.29	337	*Tetrastigma obtectum*	108.59	31.63
90	*Tetrastigma obtectum*	102.63	24.96	338	*Tetrastigma obtectum*	108.62	30.44
91	*Tetrastigma obtectum*	102.64	28.40	339	*Tetrastigma obtectum*	108.66	31.95
92	*Tetrastigma obtectum*	102.66	25.04	340	*Tetrastigma obtectum*	108.67	26.08
93	*Tetrastigma obtectum*	102.67	25.03	341	*Tetrastigma obtectum*	108.68	28.23
94	*Tetrastigma obtectum*	102.68	29.35	342	*Tetrastigma obtectum*	108.69	27.92
95	*Tetrastigma obtectum*	102.70	25.01	343	*Tetrastigma obtectum*	108.70	27.93
96	*Tetrastigma obtectum*	102.73	30.02	344	*Tetrastigma obtectum*	108.72	31.66
97	*Tetrastigma obtectum*	102.74	29.83	345	*Tetrastigma obtectum*	108.73	29.84
98	*Tetrastigma obtectum*	102.75	30.06	346	*Tetrastigma obtectum*	108.74	27.82
99	*Tetrastigma obtectum*	102.76	29.97	347	*Tetrastigma obtectum*	108.76	31.71
100	*Tetrastigma obtectum*	102.77	25.08	348	*Tetrastigma obtectum*	108.77	31.64
101	*Tetrastigma obtectum*	102.78	29.70	349	*Tetrastigma obtectum*	108.79	25.27
102	*Tetrastigma obtectum*	102.81	30.34	350	*Tetrastigma obtectum*	108.84	27.70
103	*Tetrastigma obtectum*	102.82	30.37	351	*Tetrastigma obtectum*	108.85	27.70
104	*Tetrastigma obtectum*	102.83	23.63	352	*Tetrastigma obtectum*	108.90	32.32
105	*Tetrastigma obtectum*	102.85	29.80	353	*Tetrastigma obtectum*	108.94	30.29
106	*Tetrastigma obtectum*	102.88	30.59	354	*Tetrastigma obtectum*	108.96	27.51
107	*Tetrastigma obtectum*	102.92	26.92	355	*Tetrastigma obtectum*	108.97	29.42
108	*Tetrastigma obtectum*	102.93	24.71	356	*Tetrastigma obtectum*	109.01	31.83
109	*Tetrastigma*	103.00	25.28	357	*Tetrastigma*	109.05	30.02

No.	Species	Longitude（°）	Latitude（°）	No.	Species	Longitude（°）	Latitude（°）
	obtectum				*obtectum*		
110	*Tetrastigma obtectum*	103.01	29.98	358	*Tetrastigma obtectum*	109.09	31.60
111	*Tetrastigma obtectum*	103.03	25.35	359	*Tetrastigma obtectum*	109.10	29.47
112	*Tetrastigma obtectum*	103.04	25.34	360	*Tetrastigma obtectum*	109.13	26.23
113	*Tetrastigma obtectum*	103.06	29.01	361	*Tetrastigma obtectum*	109.15	29.67
114	*Tetrastigma obtectum*	103.07	26.33	362	*Tetrastigma obtectum*	109.17	27.37
115	*Tetrastigma obtectum*	103.10	29.85	363	*Tetrastigma obtectum*	109.20	26.68
116	*Tetrastigma obtectum*	103.12	30.32	364	*Tetrastigma obtectum*	109.25	25.07
117	*Tetrastigma obtectum*	103.16	30.45	365	*Tetrastigma obtectum*	109.26	24.24
118	*Tetrastigma obtectum*	103.18	26.09	366	*Tetrastigma obtectum*	109.27	26.48
119	*Tetrastigma obtectum*	103.23	25.23	367	*Tetrastigma obtectum*	109.28	31.25
120	*Tetrastigma obtectum*	103.25	27.70	368	*Tetrastigma obtectum*	109.30	29.66
121	*Tetrastigma obtectum*	103.26	31.08	369	*Tetrastigma obtectum*	109.33	28.69
122	*Tetrastigma obtectum*	103.27	29.23	370	*Tetrastigma obtectum*	109.34	28.70
123	*Tetrastigma obtectum*	103.31	31.09	371	*Tetrastigma obtectum*	109.35	32.40
124	*Tetrastigma obtectum*	103.33	29.52	372	*Tetrastigma obtectum*	109.37	29.33
125	*Tetrastigma obtectum*	103.34	30.92	373	*Tetrastigma obtectum*	109.38	32.83
126	*Tetrastigma obtectum*	103.36	29.55	374	*Tetrastigma obtectum*	109.40	29.49
127	*Tetrastigma obtectum*	103.37	22.95	375	*Tetrastigma obtectum*	109.41	28.72
128	*Tetrastigma obtectum*	103.38	29.56	376	*Tetrastigma obtectum*	109.43	29.47
129	*Tetrastigma obtectum*	103.39	23.37	377	*Tetrastigma obtectum*	109.44	29.46
130	*Tetrastigma obtectum*	103.40	29.50	378	*Tetrastigma obtectum*	109.45	30.66

No.	Species	Longitude（°）	Latitude（°）	No.	Species	Longitude（°）	Latitude（°）
131	*Tetrastigma obtectum*	103.41	29.57	379	*Tetrastigma obtectum*	109.47	28.74
132	*Tetrastigma obtectum*	103.42	22.85	380	*Tetrastigma obtectum*	109.48	28.57
133	*Tetrastigma obtectum*	103.48	29.60	381	*Tetrastigma obtectum*	109.50	28.78
134	*Tetrastigma obtectum*	103.49	29.46	382	*Tetrastigma obtectum*	109.51	18.61
135	*Tetrastigma obtectum*	103.50	29.83	383	*Tetrastigma obtectum*	109.53	18.30
136	*Tetrastigma obtectum*	103.51	29.21	384	*Tetrastigma obtectum*	109.54	18.33
137	*Tetrastigma obtectum*	103.52	29.78	385	*Tetrastigma obtectum*	109.57	29.70
138	*Tetrastigma obtectum*	103.54	30.87	386	*Tetrastigma obtectum*	109.58	32.12
139	*Tetrastigma obtectum*	103.57	28.27	387	*Tetrastigma obtectum*	109.62	31.37
140	*Tetrastigma obtectum*	103.59	29.83	388	*Tetrastigma obtectum*	109.63	31.37
141	*Tetrastigma obtectum*	103.61	31.00	389	*Tetrastigma obtectum*	109.65	28.72
142	*Tetrastigma obtectum*	103.62	31.00	390	*Tetrastigma obtectum*	109.68	31.59
143	*Tetrastigma obtectum*	103.63	28.23	391	*Tetrastigma obtectum*	109.70	25.66
144	*Tetrastigma obtectum*	103.64	28.23	392	*Tetrastigma obtectum*	109.71	25.57
145	*Tetrastigma obtectum*	103.65	30.99	393	*Tetrastigma obtectum*	109.72	30.02
146	*Tetrastigma obtectum*	103.66	22.95	394	*Tetrastigma obtectum*	109.73	30.60
147	*Tetrastigma obtectum*	103.68	22.98	395	*Tetrastigma obtectum*	109.75	29.75
148	*Tetrastigma obtectum*	103.71	27.34	396	*Tetrastigma obtectum*	109.78	29.86
149	*Tetrastigma obtectum*	103.72	31.09	397	*Tetrastigma obtectum*	109.81	29.68
150	*Tetrastigma obtectum*	103.74	34.96	398	*Tetrastigma obtectum*	109.83	27.20
151	*Tetrastigma obtectum*	103.80	30.97	399	*Tetrastigma obtectum*	109.85	29.01
152	*Tetrastigma*	103.90	28.75	400	*Tetrastigma*	109.87	26.33

No.	Species	Longitude（°）	Latitude（°）	No.	Species	Longitude（°）	Latitude（°）
	obtectum				*obtectum*		
153	*Tetrastigma obtectum*	103.92	27.72	401	*Tetrastigma obtectum*	109.88	31.08
154	*Tetrastigma obtectum*	103.94	22.53	402	*Tetrastigma obtectum*	109.91	25.60
155	*Tetrastigma obtectum*	103.95	23.28	403	*Tetrastigma obtectum*	109.95	28.62
156	*Tetrastigma obtectum*	103.97	22.52	404	*Tetrastigma obtectum*	109.96	31.42
157	*Tetrastigma obtectum*	104.01	28.67	405	*Tetrastigma obtectum*	109.98	31.40
158	*Tetrastigma obtectum*	104.02	32.02	406	*Tetrastigma obtectum*	109.99	29.48
159	*Tetrastigma obtectum*	104.03	22.67	407	*Tetrastigma obtectum*	110.01	25.80
160	*Tetrastigma obtectum*	104.13	28.86	408	*Tetrastigma obtectum*	110.03	29.89
161	*Tetrastigma obtectum*	104.18	22.93	409	*Tetrastigma obtectum*	110.05	28.24
162	*Tetrastigma obtectum*	104.20	35.86	410	*Tetrastigma obtectum*	110.07	29.70
163	*Tetrastigma obtectum*	104.23	23.39	411	*Tetrastigma obtectum*	110.09	29.73
164	*Tetrastigma obtectum*	104.25	23.37	412	*Tetrastigma obtectum*	110.10	27.47
165	*Tetrastigma obtectum*	104.33	28.83	413	*Tetrastigma obtectum*	110.15	25.43
166	*Tetrastigma obtectum*	104.35	30.52	414	*Tetrastigma obtectum*	110.16	29.39
167	*Tetrastigma obtectum*	104.43	25.79	415	*Tetrastigma obtectum*	110.17	25.64
168	*Tetrastigma obtectum*	104.52	28.17	416	*Tetrastigma obtectum*	110.18	24.13
169	*Tetrastigma obtectum*	104.53	32.42	417	*Tetrastigma obtectum*	110.19	25.48
170	*Tetrastigma obtectum*	104.54	24.76	418	*Tetrastigma obtectum*	110.24	24.15
171	*Tetrastigma obtectum*	104.59	31.66	419	*Tetrastigma obtectum*	110.25	24.43
172	*Tetrastigma obtectum*	104.62	27.94	420	*Tetrastigma obtectum*	110.27	28.80
173	*Tetrastigma obtectum*	104.67	23.44	421	*Tetrastigma obtectum*	110.29	28.78

No.	Species	Longitude（°）	Latitude（°）	No.	Species	Longitude（°）	Latitude（°）
174	*Tetrastigma obtectum*	104.68	32.94	422	*Tetrastigma obtectum*	110.33	31.05
175	*Tetrastigma obtectum*	104.70	23.13	423	*Tetrastigma obtectum*	110.34	31.04
176	*Tetrastigma obtectum*	104.71	23.39	424	*Tetrastigma obtectum*	110.43	29.83
177	*Tetrastigma obtectum*	104.72	30.40	425	*Tetrastigma obtectum*	110.45	29.06
178	*Tetrastigma obtectum*	104.74	23.06	426	*Tetrastigma obtectum*	110.48	24.78
179	*Tetrastigma obtectum*	104.80	23.17	427	*Tetrastigma obtectum*	110.50	31.50
180	*Tetrastigma obtectum*	104.81	22.95	428	*Tetrastigma obtectum*	110.51	25.06
181	*Tetrastigma obtectum*	104.82	23.39	429	*Tetrastigma obtectum*	110.56	30.02
182	*Tetrastigma obtectum*	104.85	22.97	430	*Tetrastigma obtectum*	110.57	27.05
183	*Tetrastigma obtectum*	104.87	27.44	431	*Tetrastigma obtectum*	110.60	30.20
184	*Tetrastigma obtectum*	104.88	24.93	432	*Tetrastigma obtectum*	110.63	26.72
185	*Tetrastigma obtectum*	104.89	25.08	433	*Tetrastigma obtectum*	110.67	25.62
186	*Tetrastigma obtectum*	104.90	25.08	434	*Tetrastigma obtectum*	110.68	31.74
187	*Tetrastigma obtectum*	104.92	24.81	435	*Tetrastigma obtectum*	110.74	26.27
188	*Tetrastigma obtectum*	104.97	28.47	436	*Tetrastigma obtectum*	110.76	31.81
189	*Tetrastigma obtectum*	105.01	25.16	437	*Tetrastigma obtectum*	110.80	30.11
190	*Tetrastigma obtectum*	105.04	23.37	438	*Tetrastigma obtectum*	110.81	26.38
191	*Tetrastigma obtectum*	105.06	24.05	439	*Tetrastigma obtectum*	110.85	26.43
192	*Tetrastigma obtectum*	105.07	24.05	440	*Tetrastigma obtectum*	110.95	22.35
193	*Tetrastigma obtectum*	105.08	25.09	441	*Tetrastigma obtectum*	110.96	30.24
194	*Tetrastigma obtectum*	105.10	32.70	442	*Tetrastigma obtectum*	110.97	22.63
195	*Tetrastigma*	105.11	32.73	443	*Tetrastigma*	111.03	22.45

No.	Species	Longitude（°）	Latitude（°）	No.	Species	Longitude（°）	Latitude（°）
	obtectum				*obtectum*		
196	*Tetrastigma obtectum*	105.17	24.64	444	*Tetrastigma obtectum*	111.14	25.56
197	*Tetrastigma obtectum*	105.20	24.98	445	*Tetrastigma obtectum*	111.16	25.50
198	*Tetrastigma obtectum*	105.21	32.70	446	*Tetrastigma obtectum*	111.20	22.27
199	*Tetrastigma obtectum*	105.23	23.57	447	*Tetrastigma obtectum*	111.30	30.73
200	*Tetrastigma obtectum*	105.25	32.75	448	*Tetrastigma obtectum*	111.32	26.39
201	*Tetrastigma obtectum*	105.26	24.75	449	*Tetrastigma obtectum*	111.36	29.58
202	*Tetrastigma obtectum*	105.28	25.46	450	*Tetrastigma obtectum*	111.38	29.58
203	*Tetrastigma obtectum*	105.33	24.68	451	*Tetrastigma obtectum*	111.41	22.45
204	*Tetrastigma obtectum*	105.34	24.77	452	*Tetrastigma obtectum*	111.52	28.18
205	*Tetrastigma obtectum*	105.35	24.15	453	*Tetrastigma obtectum*	111.57	24.40
206	*Tetrastigma obtectum*	105.37	26.42	454	*Tetrastigma obtectum*	111.84	31.77
207	*Tetrastigma obtectum*	105.39	25.13	455	*Tetrastigma obtectum*	111.93	25.60
208	*Tetrastigma obtectum*	105.44	24.50	456	*Tetrastigma obtectum*	112.00	28.00
209	*Tetrastigma obtectum*	105.45	24.19	457	*Tetrastigma obtectum*	112.16	28.52
210	*Tetrastigma obtectum*	105.46	27.60	458	*Tetrastigma obtectum*	112.57	23.17
211	*Tetrastigma obtectum*	105.47	25.12	459	*Tetrastigma obtectum*	112.66	27.12
212	*Tetrastigma obtectum*	105.48	28.26	460	*Tetrastigma obtectum*	112.69	27.29
213	*Tetrastigma obtectum*	105.50	25.43	461	*Tetrastigma obtectum*	112.70	27.29
214	*Tetrastigma obtectum*	105.52	32.28	462	*Tetrastigma obtectum*	113.07	24.90
215	*Tetrastigma obtectum*	105.54	23.24	463	*Tetrastigma obtectum*	113.08	24.91
216	*Tetrastigma obtectum*	105.55	23.20	464	*Tetrastigma obtectum*	113.17	30.66

No.	Species	Longitude（°）	Latitude（°）	No.	Species	Longitude（°）	Latitude（°）
217	*Tetrastigma obtectum*	105.56	28.24	465	*Tetrastigma obtectum*	113.21	25.08
218	*Tetrastigma obtectum*	105.57	23.16	466	*Tetrastigma obtectum*	113.35	25.13
219	*Tetrastigma obtectum*	105.59	24.60	467	*Tetrastigma obtectum*	113.36	23.19
220	*Tetrastigma obtectum*	105.60	33.33	468	*Tetrastigma obtectum*	113.59	23.55
221	*Tetrastigma obtectum*	105.61	25.14	469	*Tetrastigma obtectum*	113.73	25.85
222	*Tetrastigma obtectum*	105.63	23.63	470	*Tetrastigma obtectum*	113.74	25.02
223	*Tetrastigma obtectum*	105.64	32.37	471	*Tetrastigma obtectum*	113.81	23.74
224	*Tetrastigma obtectum*	105.68	25.33	472	*Tetrastigma obtectum*	114.15	26.54
225	*Tetrastigma obtectum*	105.70	25.42	473	*Tetrastigma obtectum*	114.17	26.02
226	*Tetrastigma obtectum*	105.71	27.14	474	*Tetrastigma obtectum*	114.21	27.13
227	*Tetrastigma obtectum*	105.74	23.31	475	*Tetrastigma obtectum*	114.48	25.92
228	*Tetrastigma obtectum*	105.75	28.38	476	*Tetrastigma obtectum*	114.52	24.75
229	*Tetrastigma obtectum*	105.77	26.67	477	*Tetrastigma obtectum*	114.53	25.79
230	*Tetrastigma obtectum*	105.81	23.28	478	*Tetrastigma obtectum*	114.54	25.79
231	*Tetrastigma obtectum*	105.82	23.26	479	*Tetrastigma obtectum*	114.57	29.03
232	*Tetrastigma obtectum*	105.83	23.42	480	*Tetrastigma obtectum*	114.71	24.61
233	*Tetrastigma obtectum*	105.84	28.48	481	*Tetrastigma obtectum*	114.81	24.90
234	*Tetrastigma obtectum*	105.85	26.34	482	*Tetrastigma obtectum*	114.87	28.83
235	*Tetrastigma obtectum*	105.89	26.09	483	*Tetrastigma obtectum*	115.38	25.05
236	*Tetrastigma obtectum*	105.90	28.48	484	*Tetrastigma obtectum*	115.40	30.78
237	*Tetrastigma obtectum*	105.93	31.73	485	*Tetrastigma obtectum*	115.65	24.96
238	*Tetrastigma*	105.96	27.92	486	*Tetrastigma*	115.95	24.90

No.	Species	Longitude（°）	Latitude（°）	No.	Species	Longitude（°）	Latitude（°）
	obtectum				*obtectum*		
239	*Tetrastigma obtectum*	105.97	32.32	487	*Tetrastigma obtectum*	115.96	24.90
240	*Tetrastigma obtectum*	105.98	26.21	488	*Tetrastigma obtectum*	117.23	24.88
241	*Tetrastigma obtectum*	106.00	27.37	489	*Tetrastigma obtectum*	118.13	25.43
242	*Tetrastigma obtectum*	106.02	28.25	490	*Tetrastigma obtectum*	118.91	30.11
243	*Tetrastigma obtectum*	106.03	27.36	491	*Tetrastigma obtectum*	119.22	30.16
244	*Tetrastigma obtectum*	106.08	25.75	492	*Tetrastigma obtectum*	119.72	27.56
245	*Tetrastigma obtectum*	106.09	25.95	493	*Tetrastigma obtectum*	120.05	27.39
246	*Tetrastigma obtectum*	106.10	25.17	494	*Tetrastigma obtectum*	121.55	25.15
247	*Tetrastigma obtectum*	106.11	25.36	495	*Tetrastigma obtectum*	121.67	24.91
248	*Tetrastigma obtectum*	106.13	25.98				
1	*Tetrastigma obovatum*	95.18	29.24	48	*Tetrastigma obovatum*	103.52	30.97
2	*Tetrastigma obovatum*	95.33	29.33	49	*Tetrastigma obovatum*	103.57	28.27
3	*Tetrastigma obovatum*	97.02	28.50	50	*Tetrastigma obovatum*	103.62	31.00
4	*Tetrastigma obovatum*	97.03	29.32	51	*Tetrastigma obovatum*	103.69	28.55
5	*Tetrastigma obovatum*	97.46	28.66	52	*Tetrastigma obovatum*	103.77	22.79
6	*Tetrastigma obovatum*	97.66	24.61	53	*Tetrastigma obovatum*	103.94	22.53
7	*Tetrastigma obovatum*	98.28	25.32	54	*Tetrastigma obovatum*	103.96	22.70
8	*Tetrastigma obovatum*	98.65	25.42	55	*Tetrastigma obovatum*	103.97	22.52
9	*Tetrastigma obovatum*	98.67	27.74	56	*Tetrastigma obovatum*	103.97	24.44
10	*Tetrastigma obovatum*	99.10	23.28	57	*Tetrastigma obovatum*	103.99	23.26
11	*Tetrastigma obovatum*	99.28	23.66	58	*Tetrastigma obovatum*	104.03	28.78

No.	Species	Longitude（°）	Latitude（°）	No.	Species	Longitude（°）	Latitude（°）
12	*Tetrastigma obovatum*	100.23	25.59	59	*Tetrastigma obovatum*	104.33	28.83
13	*Tetrastigma obovatum*	100.27	21.99	60	*Tetrastigma obovatum*	104.53	32.41
14	*Tetrastigma obovatum*	100.42	23.81	61	*Tetrastigma obovatum*	104.55	32.44
15	*Tetrastigma obovatum*	100.45	21.96	62	*Tetrastigma obovatum*	105.63	23.63
16	*Tetrastigma obovatum*	100.51	21.49	63	*Tetrastigma obovatum*	106.30	26.07
17	*Tetrastigma obovatum*	100.57	21.86	64	*Tetrastigma obovatum*	106.41	28.50
18	*Tetrastigma obovatum*	100.67	24.48	65	*Tetrastigma obovatum*	106.93	27.28
19	*Tetrastigma obovatum*	100.77	22.00	66	*Tetrastigma obovatum*	107.10	29.16
20	*Tetrastigma obovatum*	100.80	22.02	67	*Tetrastigma obovatum*	107.41	26.06
21	*Tetrastigma obovatum*	100.83	24.45	68	*Tetrastigma obovatum*	107.59	23.33
22	*Tetrastigma obovatum*	100.87	22.17	69	*Tetrastigma obovatum*	107.77	27.13
23	*Tetrastigma obovatum*	100.90	22.09	70	*Tetrastigma obovatum*	108.03	32.08
24	*Tetrastigma obovatum*	101.01	24.51	71	*Tetrastigma obovatum*	108.04	32.06
25	*Tetrastigma obovatum*	101.03	22.45	72	*Tetrastigma obovatum*	108.06	32.15
26	*Tetrastigma obovatum*	101.06	22.50	73	*Tetrastigma obovatum*	108.23	27.51
27	*Tetrastigma obovatum*	101.26	21.93	74	*Tetrastigma obovatum*	108.40	27.99
28	*Tetrastigma obovatum*	101.28	21.92	75	*Tetrastigma obovatum*	108.80	28.09
29	*Tetrastigma obovatum*	101.32	21.87	76	*Tetrastigma obovatum*	109.15	29.68
30	*Tetrastigma obovatum*	101.47	21.98	77	*Tetrastigma obovatum*	109.20	28.15
31	*Tetrastigma obovatum*	101.49	21.55	78	*Tetrastigma obovatum*	109.40	29.52
32	*Tetrastigma obovatum*	101.54	21.28	79	*Tetrastigma obovatum*	109.48	29.99
33	*Tetrastigma*	101.56	21.46	80	*Tetrastigma*	109.49	28.14

No.	Species	Longitude（°）	Latitude（°）	No.	Species	Longitude（°）	Latitude（°）
	obovatum				*obovatum*		
34	*Tetrastigma obovatum*	101.57	21.48	81	*Tetrastigma obovatum*	109.56	28.07
35	*Tetrastigma obovatum*	101.77	23.59	82	*Tetrastigma obovatum*	109.74	31.86
36	*Tetrastigma obovatum*	101.78	21.63	83	*Tetrastigma obovatum*	109.78	29.08
37	*Tetrastigma obovatum*	101.99	24.07	84	*Tetrastigma obovatum*	109.85	29.00
38	*Tetrastigma obovatum*	102.38	23.13	85	*Tetrastigma obovatum*	110.09	29.73
39	*Tetrastigma obovatum*	102.44	24.91	86	*Tetrastigma obovatum*	110.16	29.39
40	*Tetrastigma obovatum*	102.62	24.98	87	*Tetrastigma obovatum*	110.33	31.05
41	*Tetrastigma obovatum*	102.66	25.04	88	*Tetrastigma obovatum*	110.34	31.04
42	*Tetrastigma obovatum*	102.74	25.14	89	*Tetrastigma obovatum*	110.68	31.74
43	*Tetrastigma obovatum*	102.83	24.49	90	*Tetrastigma obovatum*	110.95	22.35
44	*Tetrastigma obovatum*	103.09	22.79	91	*Tetrastigma obovatum*	111.02	29.69
45	*Tetrastigma obovatum*	103.23	22.78	92	*Tetrastigma obovatum*	112.90	33.90
46	*Tetrastigma obovatum*	103.39	29.49	93	*Tetrastigma obovatum*	116.70	23.37
47	*Tetrastigma obovatum*	103.39	29.55				
1	*Tetrastigma cruciatum*	98.87	26.90	43	*Tetrastigma cruciatum*	101.07	21.98
2	*Tetrastigma cruciatum*	98.88	23.55	44	*Tetrastigma cruciatum*	101.10	21.99
3	*Tetrastigma cruciatum*	98.89	26.61	45	*Tetrastigma cruciatum*	101.13	22.61
4	*Tetrastigma cruciatum*	98.98	23.37	46	*Tetrastigma cruciatum*	101.19	22.57
5	*Tetrastigma cruciatum*	98.99	23.37	47	*Tetrastigma cruciatum*	101.20	21.97
6	*Tetrastigma cruciatum*	99.22	26.19	48	*Tetrastigma cruciatum*	101.25	21.94
7	*Tetrastigma cruciatum*	99.25	23.15	49	*Tetrastigma cruciatum*	101.26	21.93

No.	Species	Longitude（°）	Latitude（°）	No.	Species	Longitude（°）	Latitude（°）
8	*Tetrastigma cruciatum*	99.38	22.20	50	*Tetrastigma cruciatum*	101.27	21.20
9	*Tetrastigma cruciatum*	99.40	23.54	51	*Tetrastigma cruciatum*	101.29	22.40
10	*Tetrastigma cruciatum*	99.46	22.25	52	*Tetrastigma cruciatum*	101.30	21.89
11	*Tetrastigma cruciatum*	99.52	22.30	53	*Tetrastigma cruciatum*	101.31	21.45
12	*Tetrastigma cruciatum*	99.61	22.64	54	*Tetrastigma cruciatum*	101.32	21.87
13	*Tetrastigma cruciatum*	99.93	22.56	55	*Tetrastigma cruciatum*	101.35	21.83
14	*Tetrastigma cruciatum*	100.22	25.58	56	*Tetrastigma cruciatum*	101.38	21.74
15	*Tetrastigma cruciatum*	100.26	22.05	57	*Tetrastigma cruciatum*	101.42	21.68
16	*Tetrastigma cruciatum*	100.27	21.99	58	*Tetrastigma cruciatum*	101.47	21.52
17	*Tetrastigma cruciatum*	100.45	21.96	59	*Tetrastigma cruciatum*	101.56	21.46
18	*Tetrastigma cruciatum*	100.47	22.38	60	*Tetrastigma cruciatum*	101.57	21.48
19	*Tetrastigma cruciatum*	100.59	23.20	61	*Tetrastigma cruciatum*	101.58	21.60
20	*Tetrastigma cruciatum*	100.60	22.25	62	*Tetrastigma cruciatum*	101.64	21.73
21	*Tetrastigma cruciatum*	100.61	22.82	63	*Tetrastigma cruciatum*	101.72	21.25
22	*Tetrastigma cruciatum*	100.66	21.58	64	*Tetrastigma cruciatum*	101.78	21.63
23	*Tetrastigma cruciatum*	100.67	22.17	65	*Tetrastigma cruciatum*	102.39	22.99
24	*Tetrastigma cruciatum*	100.68	21.58	66	*Tetrastigma cruciatum*	102.78	22.75
25	*Tetrastigma cruciatum*	100.70	23.50	67	*Tetrastigma cruciatum*	102.96	23.38
26	*Tetrastigma cruciatum*	100.72	21.67	68	*Tetrastigma cruciatum*	102.97	22.90
27	*Tetrastigma cruciatum*	100.76	21.95	69	*Tetrastigma cruciatum*	103.09	22.79
28	*Tetrastigma cruciatum*	100.80	22.01	70	*Tetrastigma cruciatum*	103.41	23.03
29	*Tetrastigma*	100.80	23.67	71	*Tetrastigma*	104.36	22.75

No.	Species	Longitude（°）	Latitude（°）	No.	Species	Longitude（°）	Latitude（°）
	cruciatum				*cruciatum*		
30	*Tetrastigma cruciatum*	100.83	22.23	72	*Tetrastigma cruciatum*	104.70	23.12
31	*Tetrastigma cruciatum*	100.86	22.70	73	*Tetrastigma cruciatum*	105.79	26.04
32	*Tetrastigma cruciatum*	100.88	22.08	74	*Tetrastigma cruciatum*	108.65	19.10
33	*Tetrastigma cruciatum*	100.89	22.25	75	*Tetrastigma cruciatum*	108.87	19.17
34	*Tetrastigma cruciatum*	100.90	22.09	76	*Tetrastigma cruciatum*	109.11	19.14
35	*Tetrastigma cruciatum*	100.91	22.08	77	*Tetrastigma cruciatum*	109.32	21.53
36	*Tetrastigma cruciatum*	100.92	22.27	78	*Tetrastigma cruciatum*	109.50	18.25
37	*Tetrastigma cruciatum*	100.93	22.17	79	*Tetrastigma cruciatum*	109.50	19.51
38	*Tetrastigma cruciatum*	100.95	23.38	80	*Tetrastigma cruciatum*	109.51	18.25
39	*Tetrastigma cruciatum*	100.97	22.66	81	*Tetrastigma cruciatum*	109.70	18.63
40	*Tetrastigma cruciatum*	101.03	22.58	82	*Tetrastigma cruciatum*	110.03	18.52
41	*Tetrastigma cruciatum*	101.05	22.60	83	*Tetrastigma cruciatum*	110.03	18.50
42	*Tetrastigma cruciatum*	101.06	22.50	84	*Tetrastigma cruciatum*	110.04	18.51
1	*Tetrastigma serrulatum*	85.29	28.85	166	*Tetrastigma serrulatum*	102.12	26.88
2	*Tetrastigma serrulatum*	85.98	27.96	167	*Tetrastigma serrulatum*	102.13	29.64
3	*Tetrastigma serrulatum*	87.42	27.87	168	*Tetrastigma serrulatum*	102.17	24.67
4	*Tetrastigma serrulatum*	87.77	28.36	169	*Tetrastigma serrulatum*	102.24	24.85
5	*Tetrastigma serrulatum*	94.58	29.56	170	*Tetrastigma serrulatum*	102.26	22.88
6	*Tetrastigma serrulatum*	94.80	29.94	171	*Tetrastigma serrulatum*	102.27	24.31
7	*Tetrastigma serrulatum*	94.91	29.99	172	*Tetrastigma serrulatum*	102.29	24.21
8	*Tetrastigma serrulatum*	94.97	30.01	173	*Tetrastigma serrulatum*	102.30	24.31

No.	Species	Longitude（°）	Latitude（°）	No.	Species	Longitude（°）	Latitude（°）
9	*Tetrastigma serrulatum*	95.10	29.22	174	*Tetrastigma serrulatum*	102.37	25.54
10	*Tetrastigma serrulatum*	95.13	29.37	175	*Tetrastigma serrulatum*	102.40	25.53
11	*Tetrastigma serrulatum*	95.18	29.24	176	*Tetrastigma serrulatum*	102.45	22.98
12	*Tetrastigma serrulatum*	95.31	29.32	177	*Tetrastigma serrulatum*	102.47	25.55
13	*Tetrastigma serrulatum*	95.33	29.33	178	*Tetrastigma serrulatum*	102.50	26.96
14	*Tetrastigma serrulatum*	95.38	29.68	179	*Tetrastigma serrulatum*	102.53	25.07
15	*Tetrastigma serrulatum*	95.41	29.42	180	*Tetrastigma serrulatum*	102.56	25.76
16	*Tetrastigma serrulatum*	95.46	29.49	181	*Tetrastigma serrulatum*	102.57	28.74
17	*Tetrastigma serrulatum*	95.48	29.64	182	*Tetrastigma serrulatum*	102.58	26.63
18	*Tetrastigma serrulatum*	95.76	29.85	183	*Tetrastigma serrulatum*	102.59	25.87
19	*Tetrastigma serrulatum*	95.77	29.87	184	*Tetrastigma serrulatum*	102.62	25.06
20	*Tetrastigma serrulatum*	96.49	29.10	185	*Tetrastigma serrulatum*	102.63	24.96
21	*Tetrastigma serrulatum*	96.78	28.72	186	*Tetrastigma serrulatum*	102.66	25.04
22	*Tetrastigma serrulatum*	97.01	28.48	187	*Tetrastigma serrulatum*	102.67	28.98
23	*Tetrastigma serrulatum*	97.02	28.50	188	*Tetrastigma serrulatum*	102.70	25.01
24	*Tetrastigma serrulatum*	97.47	28.67	189	*Tetrastigma serrulatum*	102.75	25.36
25	*Tetrastigma serrulatum*	97.66	24.61	190	*Tetrastigma serrulatum*	102.76	30.07
26	*Tetrastigma serrulatum*	97.79	24.18	191	*Tetrastigma serrulatum*	102.77	26.14
27	*Tetrastigma serrulatum*	97.80	24.20	192	*Tetrastigma serrulatum*	102.78	24.04
28	*Tetrastigma serrulatum*	97.85	24.02	193	*Tetrastigma serrulatum*	102.79	26.02
29	*Tetrastigma serrulatum*	98.31	25.40	194	*Tetrastigma serrulatum*	102.82	29.79
30	*Tetrastigma*	98.32	25.39	195	*Tetrastigma*	102.83	23.63

No.	Species	Longitude（°）	Latitude（°）	No.	Species	Longitude（°）	Latitude（°）
	serrulatum				*serrulatum*		
31	*Tetrastigma serrulatum*	98.32	28.03	196	*Tetrastigma serrulatum*	102.83	24.49
32	*Tetrastigma serrulatum*	98.33	28.08	197	*Tetrastigma serrulatum*	102.88	25.05
33	*Tetrastigma serrulatum*	98.35	27.74	198	*Tetrastigma serrulatum*	102.93	24.19
34	*Tetrastigma serrulatum*	98.40	24.51	199	*Tetrastigma serrulatum*	103.00	25.28
35	*Tetrastigma serrulatum*	98.47	27.76	200	*Tetrastigma serrulatum*	103.01	24.51
36	*Tetrastigma serrulatum*	98.53	27.71	201	*Tetrastigma serrulatum*	103.03	25.35
37	*Tetrastigma serrulatum*	98.54	25.49	202	*Tetrastigma serrulatum*	103.08	25.02
38	*Tetrastigma serrulatum*	98.56	24.81	203	*Tetrastigma serrulatum*	103.09	22.79
39	*Tetrastigma serrulatum*	98.57	27.70	204	*Tetrastigma serrulatum*	103.12	23.02
40	*Tetrastigma serrulatum*	98.57	27.80	205	*Tetrastigma serrulatum*	103.27	30.66
41	*Tetrastigma serrulatum*	98.58	27.72	206	*Tetrastigma serrulatum*	103.34	30.92
42	*Tetrastigma serrulatum*	98.58	24.43	207	*Tetrastigma serrulatum*	103.39	23.37
43	*Tetrastigma serrulatum*	98.59	24.43	208	*Tetrastigma serrulatum*	103.41	23.03
44	*Tetrastigma serrulatum*	98.61	26.06	209	*Tetrastigma serrulatum*	103.48	29.60
45	*Tetrastigma serrulatum*	98.62	27.72	210	*Tetrastigma serrulatum*	103.49	29.46
46	*Tetrastigma serrulatum*	98.62	28.01	211	*Tetrastigma serrulatum*	103.51	31.03
47	*Tetrastigma serrulatum*	98.62	26.01	212	*Tetrastigma serrulatum*	103.57	28.27
48	*Tetrastigma serrulatum*	98.63	24.53	213	*Tetrastigma serrulatum*	103.61	28.51
49	*Tetrastigma serrulatum*	98.63	27.62	214	*Tetrastigma serrulatum*	103.63	31.07
50	*Tetrastigma serrulatum*	98.64	25.98	215	*Tetrastigma serrulatum*	103.68	22.98
51	*Tetrastigma serrulatum*	98.64	26.02	216	*Tetrastigma serrulatum*	103.69	22.98

No.	Species	Longitude（°）	Latitude（°）	No.	Species	Longitude（°）	Latitude（°）
52	*Tetrastigma serrulatum*	98.66	27.74	217	*Tetrastigma serrulatum*	103.69	22.92
53	*Tetrastigma serrulatum*	98.67	27.74	218	*Tetrastigma serrulatum*	103.77	24.53
54	*Tetrastigma serrulatum*	98.68	24.58	219	*Tetrastigma serrulatum*	103.79	23.25
55	*Tetrastigma serrulatum*	98.69	24.59	220	*Tetrastigma serrulatum*	103.82	25.60
56	*Tetrastigma serrulatum*	98.72	25.96	221	*Tetrastigma serrulatum*	103.87	23.32
57	*Tetrastigma serrulatum*	98.73	25.31	222	*Tetrastigma serrulatum*	103.95	28.60
58	*Tetrastigma serrulatum*	98.74	24.95	223	*Tetrastigma serrulatum*	103.96	22.70
59	*Tetrastigma serrulatum*	98.75	24.87	224	*Tetrastigma serrulatum*	103.97	22.52
60	*Tetrastigma serrulatum*	98.76	24.93	225	*Tetrastigma serrulatum*	104.05	27.63
61	*Tetrastigma serrulatum*	98.76	24.84	226	*Tetrastigma serrulatum*	104.08	22.82
62	*Tetrastigma serrulatum*	98.76	24.83	227	*Tetrastigma serrulatum*	104.20	35.86
63	*Tetrastigma serrulatum*	98.77	24.93	228	*Tetrastigma serrulatum*	104.23	28.11
64	*Tetrastigma serrulatum*	98.77	24.83	229	*Tetrastigma serrulatum*	104.25	23.37
65	*Tetrastigma serrulatum*	98.78	27.74	230	*Tetrastigma serrulatum*	104.25	23.36
66	*Tetrastigma serrulatum*	98.81	24.94	231	*Tetrastigma serrulatum*	104.33	23.63
67	*Tetrastigma serrulatum*	98.83	23.76	232	*Tetrastigma serrulatum*	104.34	23.61
68	*Tetrastigma serrulatum*	98.84	24.50	233	*Tetrastigma serrulatum*	104.35	24.51
69	*Tetrastigma serrulatum*	98.85	25.85	234	*Tetrastigma serrulatum*	104.39	24.48
70	*Tetrastigma serrulatum*	98.86	25.84	235	*Tetrastigma serrulatum*	104.40	23.02
71	*Tetrastigma serrulatum*	98.87	26.90	236	*Tetrastigma serrulatum*	104.46	23.10
72	*Tetrastigma serrulatum*	98.91	28.49	237	*Tetrastigma serrulatum*	104.57	22.95
73	*Tetrastigma*	98.92	28.46	238	*Tetrastigma*	104.58	25.13

No.	Species	Longitude（°）	Latitude（°）	No.	Species	Longitude（°）	Latitude（°）
	serrulatum				*serrulatum*		
74	*Tetrastigma serrulatum*	98.93	24.73	239	*Tetrastigma serrulatum*	104.67	23.45
75	*Tetrastigma serrulatum*	98.95	24.55	240	*Tetrastigma serrulatum*	104.67	23.44
76	*Tetrastigma serrulatum*	98.97	25.20	241	*Tetrastigma serrulatum*	104.70	23.44
77	*Tetrastigma serrulatum*	99.24	23.48	242	*Tetrastigma serrulatum*	104.70	23.13
78	*Tetrastigma serrulatum*	99.25	23.15	243	*Tetrastigma serrulatum*	104.79	23.38
79	*Tetrastigma serrulatum*	99.26	27.11	244	*Tetrastigma serrulatum*	104.85	23.40
80	*Tetrastigma serrulatum*	99.28	27.18	245	*Tetrastigma serrulatum*	104.92	28.88
81	*Tetrastigma serrulatum*	99.29	27.18	246	*Tetrastigma serrulatum*	105.04	24.78
82	*Tetrastigma serrulatum*	99.33	25.88	247	*Tetrastigma serrulatum*	105.06	24.05
83	*Tetrastigma serrulatum*	99.46	22.73	248	*Tetrastigma serrulatum*	105.20	24.97
84	*Tetrastigma serrulatum*	99.48	27.27	249	*Tetrastigma serrulatum*	105.33	24.68
85	*Tetrastigma serrulatum*	99.51	24.21	250	*Tetrastigma serrulatum*	105.35	28.13
86	*Tetrastigma serrulatum*	99.64	24.09	251	*Tetrastigma serrulatum*	105.37	24.88
87	*Tetrastigma serrulatum*	99.67	25.96	252	*Tetrastigma serrulatum*	105.45	23.30
88	*Tetrastigma serrulatum*	99.71	27.82	253	*Tetrastigma serrulatum*	105.47	25.12
89	*Tetrastigma serrulatum*	99.72	26.51	254	*Tetrastigma serrulatum*	105.48	25.34
90	*Tetrastigma serrulatum*	99.74	24.10	255	*Tetrastigma serrulatum*	105.62	23.63
91	*Tetrastigma serrulatum*	99.79	27.55	256	*Tetrastigma serrulatum*	105.63	23.63
92	*Tetrastigma serrulatum*	99.83	23.47	257	*Tetrastigma serrulatum*	105.70	25.42
93	*Tetrastigma serrulatum*	99.83	26.00	258	*Tetrastigma serrulatum*	105.74	28.18
94	*Tetrastigma serrulatum*	99.85	27.11	259	*Tetrastigma serrulatum*	105.76	26.24

No.	Species	Longitude（°）	Latitude（°）	No.	Species	Longitude（°）	Latitude（°）
95	*Tetrastigma serrulatum*	99.93	24.58	260	*Tetrastigma serrulatum*	105.77	28.22
96	*Tetrastigma serrulatum*	99.94	25.40	261	*Tetrastigma serrulatum*	105.80	23.31
97	*Tetrastigma serrulatum*	99.95	26.87	262	*Tetrastigma serrulatum*	105.81	23.28
98	*Tetrastigma serrulatum*	99.95	26.11	263	*Tetrastigma serrulatum*	105.84	22.97
99	*Tetrastigma serrulatum*	99.96	25.67	264	*Tetrastigma serrulatum*	106.17	25.25
100	*Tetrastigma serrulatum*	99.96	25.71	265	*Tetrastigma serrulatum*	106.40	28.55
101	*Tetrastigma serrulatum*	100.03	25.60	266	*Tetrastigma serrulatum*	106.53	24.43
102	*Tetrastigma serrulatum*	100.09	23.88	267	*Tetrastigma serrulatum*	106.57	22.45
103	*Tetrastigma serrulatum*	100.11	25.65	268	*Tetrastigma serrulatum*	106.62	23.33
104	*Tetrastigma serrulatum*	100.14	25.65	269	*Tetrastigma serrulatum*	106.63	24.39
105	*Tetrastigma serrulatum*	100.17	27.10	270	*Tetrastigma serrulatum*	106.64	24.51
106	*Tetrastigma serrulatum*	100.18	26.56	271	*Tetrastigma serrulatum*	106.70	22.49
107	*Tetrastigma serrulatum*	100.20	27.00	272	*Tetrastigma serrulatum*	106.85	22.35
108	*Tetrastigma serrulatum*	100.22	25.58	273	*Tetrastigma serrulatum*	106.96	22.47
109	*Tetrastigma serrulatum*	100.23	26.88	274	*Tetrastigma serrulatum*	106.98	26.45
110	*Tetrastigma serrulatum*	100.23	25.59	275	*Tetrastigma serrulatum*	107.08	25.15
111	*Tetrastigma serrulatum*	100.24	25.59	276	*Tetrastigma serrulatum*	107.10	22.68
112	*Tetrastigma serrulatum*	100.27	27.13	277	*Tetrastigma serrulatum*	107.17	29.02
113	*Tetrastigma serrulatum*	100.36	24.88	278	*Tetrastigma serrulatum*	107.52	26.25
114	*Tetrastigma serrulatum*	100.43	24.81	279	*Tetrastigma serrulatum*	107.59	23.33
115	*Tetrastigma serrulatum*	100.44	25.15	280	*Tetrastigma serrulatum*	107.60	25.05
116	*Tetrastigma*	100.45	21.97	281	*Tetrastigma*	107.76	25.08

No.	Species	Longitude（°）	Latitude（°）	No.	Species	Longitude（°）	Latitude（°）
	serrulatum				*serrulatum*		
117	*Tetrastigma serrulatum*	100.45	21.96	282	*Tetrastigma serrulatum*	107.83	25.16
118	*Tetrastigma serrulatum*	100.50	24.76	283	*Tetrastigma serrulatum*	107.97	26.58
119	*Tetrastigma serrulatum*	100.52	27.00	284	*Tetrastigma serrulatum*	108.07	26.38
120	*Tetrastigma serrulatum*	100.54	26.90	285	*Tetrastigma serrulatum*	108.08	26.38
121	*Tetrastigma serrulatum*	100.58	25.83	286	*Tetrastigma serrulatum*	108.17	26.38
122	*Tetrastigma serrulatum*	100.62	24.43	287	*Tetrastigma serrulatum*	108.28	26.44
123	*Tetrastigma serrulatum*	100.64	24.42	288	*Tetrastigma serrulatum*	108.40	28.00
124	*Tetrastigma serrulatum*	100.66	24.29	289	*Tetrastigma serrulatum*	108.43	25.67
125	*Tetrastigma serrulatum*	100.67	24.48	290	*Tetrastigma serrulatum*	108.52	25.93
126	*Tetrastigma serrulatum*	100.70	26.50	291	*Tetrastigma serrulatum*	108.64	27.91
127	*Tetrastigma serrulatum*	100.71	24.28	292	*Tetrastigma serrulatum*	108.66	25.22
128	*Tetrastigma serrulatum*	100.71	24.62	293	*Tetrastigma serrulatum*	108.68	25.23
129	*Tetrastigma serrulatum*	100.73	24.48	294	*Tetrastigma serrulatum*	108.70	27.92
130	*Tetrastigma serrulatum*	100.75	26.68	295	*Tetrastigma serrulatum*	108.79	25.27
131	*Tetrastigma serrulatum*	100.80	22.01	296	*Tetrastigma serrulatum*	108.87	28.07
132	*Tetrastigma serrulatum*	100.80	22.02	297	*Tetrastigma serrulatum*	108.90	24.78
133	*Tetrastigma serrulatum*	100.83	24.45	298	*Tetrastigma serrulatum*	109.10	27.63
134	*Tetrastigma serrulatum*	100.85	27.28	299	*Tetrastigma serrulatum*	109.18	26.67
135	*Tetrastigma serrulatum*	100.94	24.50	300	*Tetrastigma serrulatum*	109.20	28.17
136	*Tetrastigma serrulatum*	101.00	24.51	301	*Tetrastigma serrulatum*	109.20	28.15
137	*Tetrastigma serrulatum*	101.02	24.54	302	*Tetrastigma serrulatum*	109.25	25.07

No.	Species	Longitude（°）	Latitude（°）	No.	Species	Longitude（°）	Latitude（°）
138	*Tetrastigma serrulatum*	101.08	26.01	303	*Tetrastigma serrulatum*	109.30	30.02
139	*Tetrastigma serrulatum*	101.10	26.09	304	*Tetrastigma serrulatum*	109.31	25.28
140	*Tetrastigma serrulatum*	101.11	26.16	305	*Tetrastigma serrulatum*	109.40	29.52
141	*Tetrastigma serrulatum*	101.20	25.47	306	*Tetrastigma serrulatum*	109.43	29.47
142	*Tetrastigma serrulatum*	101.23	25.95	307	*Tetrastigma serrulatum*	109.50	28.77
143	*Tetrastigma serrulatum*	101.28	27.93	308	*Tetrastigma serrulatum*	109.52	28.83
144	*Tetrastigma serrulatum*	101.36	24.30	309	*Tetrastigma serrulatum*	109.59	25.64
145	*Tetrastigma serrulatum*	101.39	24.42	310	*Tetrastigma serrulatum*	109.67	24.94
146	*Tetrastigma serrulatum*	101.44	24.20	311	*Tetrastigma serrulatum*	109.71	29.58
147	*Tetrastigma serrulatum*	101.51	29.00	312	*Tetrastigma serrulatum*	109.72	30.02
148	*Tetrastigma serrulatum*	101.54	24.04	313	*Tetrastigma serrulatum*	109.75	25.26
149	*Tetrastigma serrulatum*	101.55	25.03	314	*Tetrastigma serrulatum*	109.78	29.08
150	*Tetrastigma serrulatum*	101.63	24.70	315	*Tetrastigma serrulatum*	109.83	25.20
151	*Tetrastigma serrulatum*	101.64	27.06	316	*Tetrastigma serrulatum*	109.88	25.98
152	*Tetrastigma serrulatum*	101.67	26.07	317	*Tetrastigma serrulatum*	109.89	29.02
153	*Tetrastigma serrulatum*	101.74	24.22	318	*Tetrastigma serrulatum*	109.96	28.25
154	*Tetrastigma serrulatum*	101.77	23.59	319	*Tetrastigma serrulatum*	109.97	29.22
155	*Tetrastigma serrulatum*	101.88	29.59	320	*Tetrastigma serrulatum*	110.05	28.24
156	*Tetrastigma serrulatum*	101.91	23.98	321	*Tetrastigma serrulatum*	110.09	28.66
157	*Tetrastigma serrulatum*	101.96	24.67	322	*Tetrastigma serrulatum*	110.16	29.40
158	*Tetrastigma serrulatum*	101.96	30.06	323	*Tetrastigma serrulatum*	110.22	22.89
159	*Tetrastigma*	101.97	27.04	324	*Tetrastigma*	110.55	29.35

No.	Species	Longitude（°）	Latitude（°）	No.	Species	Longitude（°）	Latitude（°）
	serrulatum				*serrulatum*		
160	*Tetrastigma serrulatum*	101.98	23.93	325	*Tetrastigma serrulatum*	110.57	30.09
161	*Tetrastigma serrulatum*	101.99	24.07	326	*Tetrastigma serrulatum*	110.68	31.74
162	*Tetrastigma serrulatum*	102.00	23.60	327	*Tetrastigma serrulatum*	110.95	22.35
163	*Tetrastigma serrulatum*	102.01	24.65	328	*Tetrastigma serrulatum*	111.03	22.45
164	*Tetrastigma serrulatum*	102.09	25.65	329	*Tetrastigma serrulatum*	116.70	23.37
165	*Tetrastigma serrulatum*	102.10	24.68				

Table S4: Description of 23 candidate predictors used in MaxEnt model under different climate scenarios.

Types	Variable	Description	Unit
Bioclimatic	Bio1	Annual mean temperature	℃
	Bio2	Mean diurnal range (mean of monthly (max temp–min temp))	℃
	Bio3	Isothermality (Bio2/Bio7) (× 100)	-
	Bio4	Temperature seasonality (standard deviation × 100)	-
	Bio5	Max temperature of warmest month	℃
	Bio6	Min temperature of coldest month	℃
	Bio7	Temperature annual range (Bio5 - Bio6)	℃
	Bio8	Mean temperature of wettest quarter	℃
	Bio9	Mean temperature of driest quarter	℃
	Bio10	Mean temperature of warmest quarter	℃
	Bio11	Mean temperature of coldest quarter	℃
	Bio12	Annual precipitation	mm
	Bio13	Precipitation of wettest month	mm
	Bio14	Precipitation of driest month	mm
	Bio15	Precipitation seasonality (coefficient of variation)	-
	Bio16	Precipitation of wettest quarter	mm
	Bio17	Precipitation of driest quarter	mm
	Bio18	Precipitation of warmest quarter	mm
	Bio19	Precipitation of coldest quarter	mm
Topographic	Aspect	-	-
	Elevation	-	m
	Slope	-	°
Anthropogenic	HI	Human influence	-
